# Synthesis and characterization of macrodiols and non-segmented poly(ester-urethanes) (PEUs) derived from α,ω-hydroxy telechelic poly(ε-caprolactone) (HOPCLOH): effect of initiator, degree of polymerization, and diisocyanate†

**DOI:** 10.1039/d4ra03951c

**Published:** 2024-08-27

**Authors:** Miriam P. Barrera-Nava, Rodrigo Navarro, Ángel Marcos-Fernández, José E. Báez

**Affiliations:** a Department of Chemistry, University of Guanajuato (UG) Noria Alta S/N 36050 Guanajuato Gto Mexico jebaez@ugto.mx; b Institute of Polymer Science and Technology, CSIC C/Juan de la Cierva No. 3 28006 Madrid Spain

## Abstract

Nine different macrodiols derived from α,ω-hydroxy telechelic poly(ε-caprolactone) (HOPCLOH) were prepared by ring-opening polymerization of ε-caprolactone (CL) using three linear aliphatic diols (HO–(CH_2_)_*n*_–OH, where *n* = 4, 8, and 12) as initiators and catalyzed by ammonium decamolybdate (NH_4_)_8_[Mo_10_O_34_]. The crystallization temperature (*T*_c_) and crystallinity (*x*_i_) were relatively high for HOPCLOH species with a long aliphatic chain [–(CH_2_)_12_–] in the oligoester. Also, HOPCLOH was the precursor of twenty-seven different poly(ester-urethanes) (PEUs) with various degrees of polymerization (DP) of HOPCLOH and three types of diisocyanates such as 1,6-hexamethylene diisocyanate (HDI), methylene diphenyl diisocyanate (MDI), and 4,4′-methylenebis (cyclohexyl isocyanate) (HMDI). HOPCLOH exhibited the melting temperature (*T*_m_) and crystallinity (*x*_i_) with a proportional dependency to the degree of polymerization (DP). PEUs showed significant thermal and mechanical properties, which had a direct correlation in terms of the type of DP and diisocyanate. PEUs derived from HDI *versus* MDI or HMDI exhibited an apparent effect where aliphatic diisocyanate (HDI) induced a significant *x*_i_ with respect to aromatic and cyclic diisocyanate (MDI or HMDI). The profile of PEUs films according to mechanical properties is mainly a plastic behavior. The chemical nature and properties of HOPCLOH and PEUs were characterized by NMR, FT-IR, GPC, MALDI-TOF, DSC, and mechanical properties.

## Abstract 2

This study explores three different factors such as the initiator, degree of polymerization and diisocyanate on the properties of macrodiols and poly(ester-urethanes) (PEUs).

## Graphical abstract

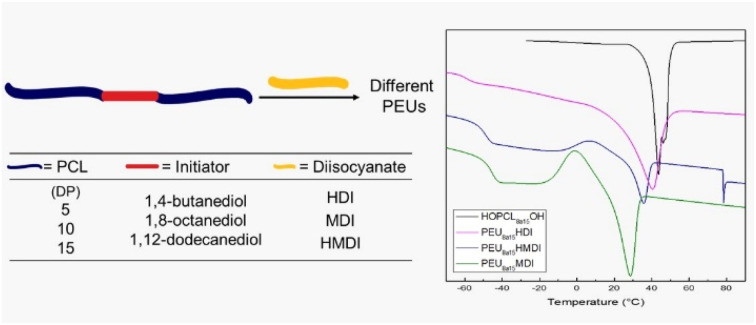

## Introduction

Poly(ε-caprolactone) (PCL) is a biodegradable aliphatic polyester [–CO–(CH_2_)_5_–O–]_n_ generally synthesized by ring-opening polymerization (ROP) of ε-caprolactone (CL).^1,2^ PCL is a hydrophobic and semi-crystalline polyester whose crystallinity tends to decrease as its molecular weight increases. The characteristics of PCL, such as solubility, low melting point (59–64 °C), biodegradability, and mixture compatibility, have stimulated extensive research into its potential application in the biomedical field^3^ as a controlled drug delivery vehicle,^4^ porous and fibrous scaffolds,^5^ or implants.^6^

Ring-opening polymerization (ROP) of CL is the common route to synthesize PCL, where a metallic or non-metallic catalyst is used, and an alcohol or amine is employed as an initiator.^7,8^ The main limitation to defining the type of architecture of the PCL produced is the initiator; for example, primary alcohols (R–OH) can result in monofunctional PCLs with an α-hydroxyl-ω-alkyl terminal group (R–PCL–OH)^9,10^ and diols (HO–R–OH) are suitable initiators to obtain α, ω-hydroxyl telechelic PCLs (HO–PCL–R–PCL–OH) (HOPCLOH),^11,12^ and these α, ω-hydroxyl telechelic PCLs can be chemically modified into analogues.^13^

α,ω-Telechelic macrodiols such as HOPCLOH can react with a diisocyanate group (OCN–R–NCO) to produce segmented amphiphilic polyurethanes,^14^ polyurethanes acrylates,^15^ poly(ester-urethane-amide),^16^ poly(ester-urethane) with shape-memory application,^17^ and poly(ester-urethane) (PEU) from miktoarm star copolymer,^18^ to mention a few. Thermoplastic polyurethanes are characterized by their elasticity, high abrasion, wear, and oxygen resistance, including resistance to oxidizing agents that can cause material breakdown or loss of mechanical properties over time. Another distinctive feature of these thermoplastic polyurethanes is the preservation of mechanical properties at different temperatures, which is why this type of polymer has been incorporated into the area of biomaterials, where PEUs as biodegradable materials derived from PCL,^19^ PDLLA,^20^ PLA,^21^ and some copolymers have been previously reported.^22^

The most common aliphatic diisocyanates used to obtain PEUs derived from HOPCLOH are 1,6-hexamethylene diisocyanate (HDI),^23,24^ and 4,4′-methylene dicyclohexyl diisocyanate (HMDI).^25,26^ On the other hand, in the case of aromatic diisocyanates, 2,4-toluene diisocyanate (TDI)^27^ and 4,4′-diphenylmethane diisocyanate (MDI) are the most commonly used.^28^ Diisocyanates play an important role in affecting the mechanical properties of polyurethane materials.^29,30^ The geometry of diisocyanates (aliphatic, cycloaliphatic, and aromatic) greatly influences the tensile strength and hardness of polyurethane materials.^31–33^

Polyurethanes synthesized from biodegradable diols have a potential application in biomaterials,^34^ tissue engineering,^35–37^ drug transport,^38^ regeneration membranes,^39^ and sustainable materials.^40^ Polyurethanes based on PCL used as memory foam have been prepared so that they have shown improvement in their properties, such as biodegradability and biocompatibility.^41–43^

**Scheme 1:**
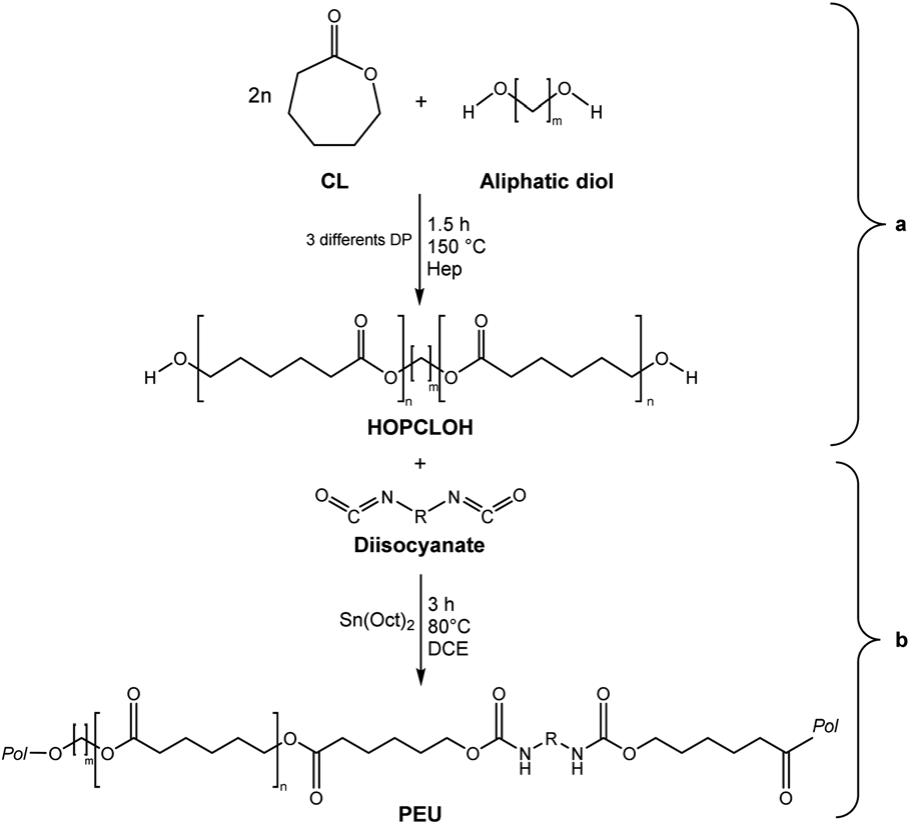
Synthesis of (a) α,ω-hydroxy telechelic poly(ε-caprolactone) (HOPCLOH), where *m* = 4, 8 and 12, with three different degrees of polymerization (DP) of 5,10 and 15. Followed by the synthesis of (b) poly(ester-urethane) (PEU) derived from HOPCLOH, where R corresponds to the central segment of the diisocyanate: 1,6-hexamethylene diisocyanate (HDI), methylene diphenyl diisocyanate (MDI), and 4,4′-methylenebis (cyclohexyl isocyanate) (HMDI).

In this work, we explored three different factors (Table 1) in the synthesis of PEUs, such as: (1) the type of diol [HO–R–OH, where R = (CH_2_)_*m*_ and *m* = 4, 8, and 12] used as an initiator in the ROP of CL to obtain the HOPCLOH (Scheme 1a); (2) the degree of polymerization (DP) of HOPCLOH, where DP = 5, 10, and 15; and (3) the variation of diisocyanates (HDI, MDI, and HMDI) (Scheme 1b). HOPCLOH and PEUs were characterized by different analytical techniques to visualize their chemical nature and understand the three factors previously described.

**Table 1:** Chemical species involved in this work: the linear aliphatic diols [HO(CH_2_)_*m*_OH, where *m* = 4, 8, and 12] used as initiators in the ROP of CL, the poly(ε-caprolactone) diols (HOPCLOH) obtained from this initiators (where *n* = 5,10, and 15; relating to de degree of polymerization), and the three diisocyanates employed for the preparation of poly(ester-urethanes) (PEUs) derived from poly(ε-caprolactone) diols (HOPCLOH)

Initiator	Macrodiol	Diisocyanate
1,4-Butanediol 1,4-BD	HOPCL_4_OH	HDI
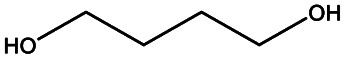	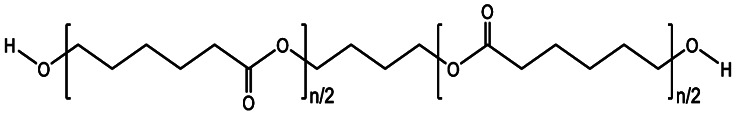	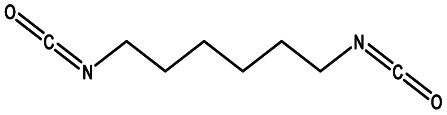
1,8-Octanediol 1,8-OD	HOPCL_8_OH	MDI
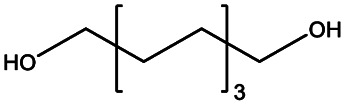	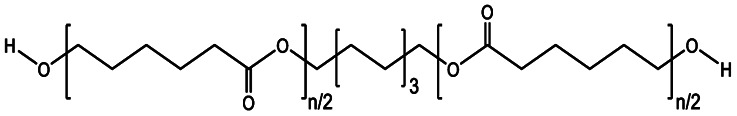	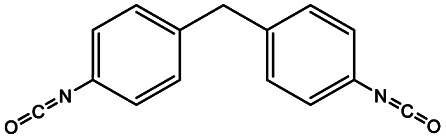
1,12-Dodecanediol 1,12-DD	HOPCL_12_OH	HMDI
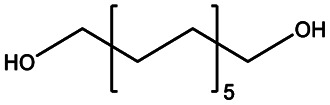	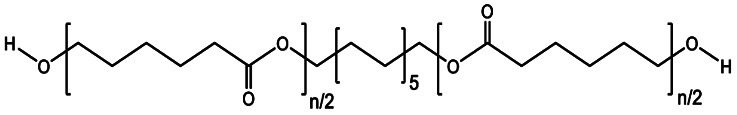	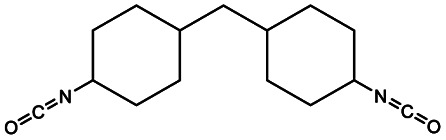

## Experimental

### Materials

ε-Caprolactone (CL) was supplied by Aldrich Chemical Co., dried over calcium hydride (CaH_2_) for 24 h and distilled under reduced pressure before use. 1,4-Butanediol, 1,8-octanediol, 1,12-dodecanediol, tin(ii) 2-ethylhexanoate Sn(Oct)_2_, 1,6-diisocyanatehexane (HDI), 4,4′-methylene dicyclohexyl diisocyanate (HMDI), methylene diphenyl diisocyanate (MDI), 1,2-dichloroethane (DCE), and ammonium heptamolybdate (NH_4_)_8_[Mo_10_O_34_]·4H_2_O (Hep) were purchased from Aldrich Chemical Co. and used without further purification.

### Synthesis of α,ω-hydroxy telechelic poly(ε-caprolactone) (HOPCL_4a10_OH or macrodiol) by (NH_4_)_8_[Mo_10_O_34_] as a catalyst and HO(CH_2_)_4_OH as an initiator

In a dried 25 mL round-bottom flask, ε-caprolactone (CL) (65 mmol, 7.419 g), ammonium heptamolybdate tetrahydrate (NH_4_)_6_[Mo_7_O_24_]·4H_2_O (Hep, 2.42 × 10^−3^ mmol, 3 mg), and 1,4-butanediol (6.5 mmol, 580 mg) were added and heated to reflux by stirring them in an oil bath at 150 °C for 1.5 h (molar ratio CL/Hep = 26 800 and CL/1,4-butanediol = 10). By thermal decomposition *in situ* of ammonium heptamolybdate (NH_4_)_6_[Mo_7_O_24_], ammonium decamolybdate (NH_4_)_8_[Mo_10_O_34_] was obtained in the solid-state [8]. Conversion and number-average molecular weight (*M*_n_) were monitored by ^1^H NMR. Once the reaction time was over, an aliquot of the crude reaction was dissolved in CDCl_3_ and analyzed by ^1^H NMR without purification. The peaks at 2.29 [–CH_2_–O–, *I*_pol_, repetitive unit of CL] and 3.63 [(–CH_2_–OH)_2_, *I*_ter_, terminal group or end group] in the ^1^H NMR spectrum were used to calculate the *M*_n_ in two steps: (1) degree of polymerization (DP). DP_(NMR)_ = (*I*_pol_ ÷ *I*_ter_) × 2. *I*_pol_ and *I*_ter_ corresponded to the integrals of the methylenes obtained by ^1^H NMR from the polyester [CH_2_–O] and α,ω-hydroxy [(–CH_2_–OH)_2_] terminal group peaks, respectively, and the ×2 was due to the bifunctionality of the polymer, α,ω-hydroxyl telechelic PCL. (2) Number-average molecular weight (*M*_n_). *M*_n_ (NMR) = (MW(CL)) (DP_(NMR)_) + MW (diol), where MW was the molecular weight of the repetitive unit (CL), and diol (1,4-butanediol, 1,8-octanediol or 1,12-dodecanediol), respectively; DP (NMR) was earlier calculated in step (1). Table 2 shows the number-average molecular weight (*M*_n_) obtained by ^1^H NMR, MALDI-TOF, and GPC, where the *M*_n_ calculated was similar to the values of *M*_n_ obtained by ^1^H NMR and MALDI-TOF.

**Table 2:** Poly(ε-caprolactone) diols (HOPCLOH) prepared using linear aliphatic diols [HO(CH_2_)_*m*_OH, where *m* = 4, 8, and 12] as initiators in the ROP of CL^a^^,^^b^

Sample	Initiator	Alkyl c^,^^d^(%)	PCL c(%)	DP (calcd)	DP (NMR)^c^	*M*_n_ (calcd)^e^	*M*_n_ (NMR)^c^^,^^f^	*M*_n_ (MALDI)^g^	*M*_n_ (GPC)^h^	Conv. c(%)
HOPCL_4a5_OH	HO(CH_2_)_4_OH	16	84	5	4.9	650	570	—	1868	98
HOPCL_4a10_OH	HO(CH_2_)_4_OH	8	92	10	10.0	1230	1120	1254	2575	98
HOPCL_4a15_OH	HO(CH_2_)_4_OH	5	95	15	14.7	1760	1660	—	3300	99
HOPCL_8a5_OH	HO(CH_2_)_8_OH	23	77	5	5.0	710	640	—	1819	99
HOPCL_8a10_OH	HO(CH_2_)_8_OH	12	88	10	10.1	1300	1210	1314	2585	99
HOPCL_8a15_OH	HO(CH_2_)_8_OH	9	91	15	14.0	1750	1630	—	3184	99
HOPCL_12a5_OH	HO(CH_2_)_12_OH	29	71	5	5.0	770	690	—	1849	99
HOPCL_12a10_OH	HO(CH_2_)_12_OH	16	84	10	10.1	1350	1250	1366	2651	99
HOPCL_12a15_OH	HO(CH_2_)_12_OH	11	89	15	15.0	1910	1780	—	3716	99

a HOPCLOH, α,ω-hydroxy telechelic poly(ε-caprolactone); ROP, ring-opening polymerization; CL, ε-caprolactone; DP, degree of polymerization; PCL, poly(ε-caprolactone).

b Polymerization at 150° for 90 min with 65 mmol of CL, three CL/initiator molar ratios of 5,10, and 15 were used in the experiments (DP (calcd) = 5, 10, or 15) and ammonium decamolybdate as a catalyst. HOPCLOH = HO–PCL–OH or HO–(CH_2_)_*m*_–PCL–OH, where *m* = 4, 8, and 12.

c Determined by ^1^H NMR in CDCl_3_.

d Obtained from the equation alkyl (%) = (MW_initiator_/*M*_n_ (NMR)) × 100, where MW_initiator_ is the molecular weight of initiator or alkyl diol (HOROH).

e Obtained from the equation *M*_n_ (calcd) = (MW (CL))(mmol CL/mmol ROH) + MW (HOROH), where MW is the molecular weight of ε-caprolactone monomer or aliphatic diol (HOROH).

f Obtained from the equation *M*_n_ (NMR) = (DP (PCL) × MW (CL)) + MW (ROH), where MW is the molecular weight of ε-caprolactone monomer or aliphatic diol (HOROH).

g Calculated by MALDI-TOF.

h Determined by GPC results.

*M*_n_ (calcd) = 1,230, *M*_n_ (NMR) = 1120 (conv. = 98%), *M*_n_ (GPC) = 2,575, *M*_w_/*M*_n_ = 1.33, *M*_n_ (MALDI) = 1254. IR (cm^−1^) 3434 (*ν*, OH, PCL), 2944 (*ν*_as_, CH_2_, PCL), 2865 (*ν*_s_, CH_2_, PCL), 1723 (*ν*, C=O, PCL), 1471 (*δ*_s_, CH_2_, PCL), 1164 (*ν*_as_, C–(C=O)–O, PCL), 1044 (*ν*_as_, O–C–C, PCL), 732 (*ρ*, CH_2_, PCL). NMR data for HOPCL_4a10_OH. ^1^H NMR (400 MHz, CDCl_3_, ppm): *δ* 4.05 (*t*, 2H, [CH_2_O], CL and 1,4-But), 3.63 (t, 4H, [(CH_2_OH)_2_], CL and But), 2.29 (t, 2H, (CH_2_–CO–O–), CL), 1.64 (m, 4H, [(CH_2_)_2_], CL), 1.55 (t, 4H, [(CH_2_)_2_], But), 1.37 (q, 2H, [CH_2_], CL). ^13^C NMR (100 MHz, CDCl_3_, ppm): …—O–CH^f^_2_–CH^h^_2_–CH^h^_2_–CH^g^_2_–O–[C(=O)^f^–CH^a^_2_–CH^b^_2_–CH^c^_2_–CH^d^_2_–CH^e^_2_–O]_*n*−1_–C(=O)^f^′–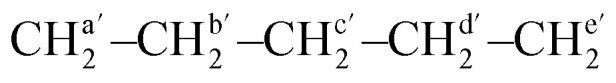
–OH: *δ* 173.74 (f′), 173.58 (f), 64.14 (g), 63.79 (e), 62.58 (e′), 34.22 (a′), 34.11 (a), 32.32 (d′), 29.15 (h), 28.34 (d), 25.52 (b), 25.30 (c′), 24.68 (b′), 24.57 (c).

### Synthesis of poly(ester-urethane) (PEU_4a10_A) derived of poly(ε-caprolactone) diol (HOPCL_4a10_OH) and 1,6-hexamethylene diisocyanate (HDI)

The reaction was carried out in a 25 mL round-bottom flask previously dried, 2.0 g (1.787 mmol) of HOPCL_4a10_OH [*M*_n_ (NMR) = 1120] was added [it was assumed that 8% of unreacted diol (HO–R–OH) was present in polymer samples with DP = 10 and DP = 15, and in the case of DP = 5 a 15% of unreacted diol (HO–R–OH) was presumed, so this fraction was contemplated in the calculation to obtain the molecular weight of the macrodiol]. In a previous contribution,^12^ unreacted diol (diethylene glycol) (DEG) was detected, according to previous results the macrodiol or polyol called HOPCLOH had a significant amount of unreacted initiator DEG when the degree of polymerization (DP) is lower, and this percentage is decreasing to relatively higher DP. In this sense, in this contribution the value of unreacted diol was 15% for lower DP (5) and 8% for medium and high DP (10 and 15). Subsequently, 333 mg (1.985 mmol) of HDI [HOPCL_4a10_OH:HDI molar ratio = 1 : 1.1] was added and dissolved in 8 mL of 1,2-dichloroethane (DCE) [0.21 M with respect to HOPCL_4a10_OH], and tin(ii) 2-ethylhexanoate [Sn(Oct)_2_] was added as catalyst [1 wt%, 32 mg ∼ 3 drops]. Then, the flask was placed in an oil bath at 80 °C for 3 hours. After the reaction time, a PEU_4a10_A film was obtained by casting at room temperature on a leveled Teflon surface within a fume cupboard covered with a conical funnel to protect it from dust and allow a slow solvent evaporation for 12 h. After, the PEU film was released and dried under a vacuum. Following the same methodology, 27 different PEUs were synthesized.

IR (cm^−1^): 3322 (*ν*, N–H, urethane), 2933 (*ν*_as_, CH_2_, PCL), 2859 (*ν*_s_, CH_2_, PCL), 1728 (*ν*, C=O, PCL), 1685 (*ν*, C=O, urethane), 1534 (*δ*, N–H, urethane), 1463 (*δ*_s_, CH_2_, PCL), 1161 (*ν*_as_, C–(C=O)–O, PCL), 1095 (*ν*_as_, O–C–C, PCL), 733 (*ρ*, CH_2_, PCL). NMR data for PEU_4a10_A. ^1^H NMR (400 MHz, CDCl_3_, ppm): *δ* 4.73 (s, 1H, [NH], urethane), 4.05 (t, 2H, [CH_2_O], CL and 1,4-But), 3.13 (t, 4H, [(CH_2_OCONH)_2_], urethane), 2.29 (t, 2H, (–CH_2_–CO–O–), CL), 1.63 (m, 4H, [(CH_2_)_2_], CL), 1.63 (t, 2H, [CH_2_], But), 1.48 (t, 2H, [CH_2_], urethane), 1.37 (q, 2H, [CH_2_], CL and But). ^13^C NMR (100 MHz, CDCl_3_, ppm): …—O–CH^f^_2_–CH^h^_2_–CH^h^_2_–CH^g^_2_–O–[C(=O)^f^–CH^a^_2_–CH^b^_2_–CH^c^_2_–CH^d^_2_–CH^e^_2_–O]_*n*−1_–C(=O)^f^–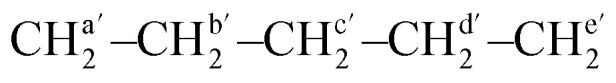
–O–C(=O)^i^–NH–CH^j^_2_–CH^k^_2_–CH^l^_2_–CH^l^_2_–CH^k^_2_–CH^j^_2_–NH: *δ* 173.65 (f), 156.86 (i), 64.65 (e′), 64.25 (g), 63.90 (e), 40.77 (j), 34.16 (a′), 34.11 (a), 29.91 (h), 28.75 (l), 28.34 (d), 25.52 (b), 25.31 (c′), 24.63 (b′), 24.57 (c).

All macromolecular species were named according to the next indications: macrodiols were named as follows; HOPCL_4a10_OH: “4” symbolizes the size of the initiator (such as 1,4-butanediol), “a” denotes aliphatic diol, and “10” represents the ε-caprolactone (CL) degree of polymerization (DP). For PEUs; PEU_4a10_A, B, and C: “A, B, and C” stand for the various diisocyanate types that were utilized (A for HDI, B for MDI, and C for HMDI); “4a10” refers to the matching macrodiol, HOPCL_4a10_OH as product (Table 2) or precursor of PEU_4a10_A (Table 4). To visualize the pattern of samples, see Tables S1 and S2.†

### Characterization methods

Nuclear magnetic resonance (NMR). ^1^H and ^13^C NMR were recorded at room temperature on a Varian Inova or Mercury 400 MHz (400 MHz ^1^H and 100 MHz ^13^C). CDCl_3_ was used as a solvent, and all spectra were referenced to the residual solvent CDCl_3_ [*δ* (ppm) 7.26 (^1^H) and 77.0 (^13^C)]. Fourier Transform Infrared Spectroscopy (FT-IR). HOPCLOHs and PEUs films were recorded with an attenuated total reflectance spectroscopy (ATR) accessory in a PerkinElmer Spectrum One FT-IR spectrometer. Differential Scanning Calorimetry (DSC). Thermograms were performed in a Mettler Toledo DSC822^e^ instrument. Three scans were obtained with two heating (25–80 °C and −90–80 °C) and one cooling (80–−90 °C) between them, at a rate of 10 °C min^−1^ and under a nitrogen purge. Gel permeation chromatography (GPC). The case for HOPCLOH: GPC measurements were determined using a Waters gel permeation chromatograph equipped with a Waters 1515 isocratic high-performance liquid chromatography (HPLC) pump and a Waters 2414 refractive index (RI) detector. A set of three Waters columns conditioned at 35 °C were used to elute samples at a flow rate of 1 mL min^−1^ of HPLC grade tetrahydrofuran (THF). Polystyrene standards (polymer laboratories) were used for calibration.

Matrix-assisted laser desorption ionization time-of-flight (MALDI-TOF). MALDI-TOF spectra were recorded in the linear mode by using a Voyager DE-PRO time-of-flight mass spectrometer (Applied Biosystems) equipped with a nitrogen laser emitting at *λ* = 337 nm with a 3 ns pulse width and working in positive ion mode and delayed extraction. A high acceleration voltage of 20 kV was employed. 2,5-Dihydroxybenzoic acid (DHB) at a concentration of 10 mg mL^−1^ in acetonitrile was used as a matrix. Samples were dissolved in acetonitrile and mixed with the matrix at a molar ratio of approximately 1 : 100. Mechanical properties. The mechanical properties were measured in an MTS testing machine equipped with a 100 N load cell. Type 3 dumbbell test pieces (according to ISO 37) were cut from the films. A crosshead speed of 200 mm min^−1^ was used. The strain was measured from crosshead separation and referred to a 12 mm initial length. At least four samples were evaluated for each PEU.

## Results and discussion

### Part 1. α,ω-hydroxy telechelic poly(ε-caprolactone) (HOPCLOH) using diols as initiators

**Fig. 1:**
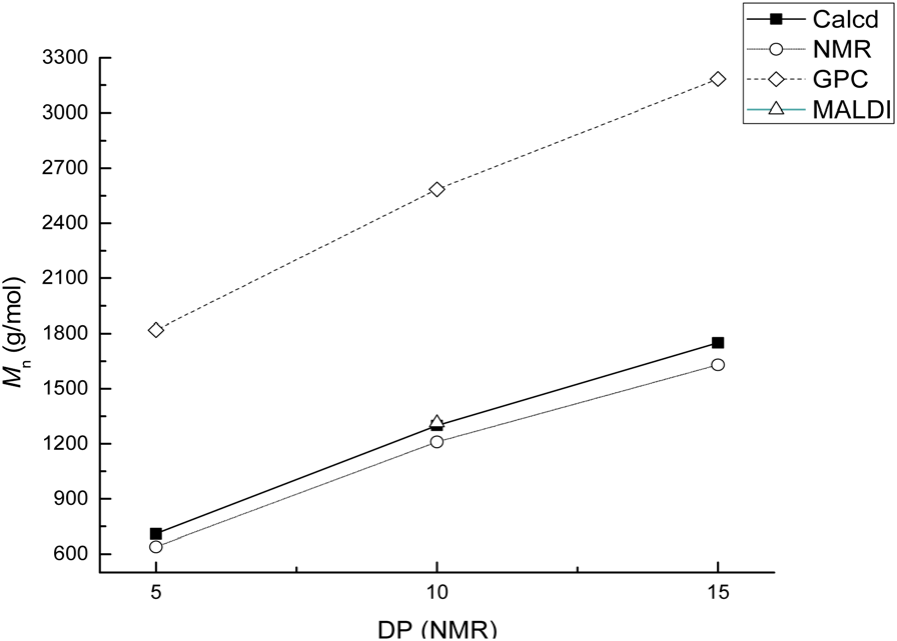
Molecular weight (*M*_n_) of poly(ε-caprolactone) diol (HOPCLOH) prepared using the linear aliphatic diol HO(CH_2_)_8_OH as an initiator in the ROP of CL with degree polymerization (DP) determined by ^1^H NMR in CDCl_3_. This data was obtained by NMR, GPC, and MALDI-TOF and compared with the calculated *M*_n_.

A series of α,ω-hydroxy telechelic poly(ε-caprolactone)s (HOPCLOHs) (Scheme 1a) were synthesized by ring-opening polymerization (ROP) of ε-caprolactone (CL) *via* bulk polymerization with an (NH_4_)_8_[Mo_10_O_34_] as a catalyst, in the presence of three different aliphatic diols as initiators [HO–(CH_2_)_*m*_–OH, where *m* = 4, 8 and 12] and obtaining a family of HOPCLOH [HO–PCL–O–(CH_2_)_*m*_–O–PCL–OH, *m* = 4, 8 and 12, with a degree of polymerization (DP) of 5, 10 and 15] (Table S1†). The purpose in the preparation of these species is to understand the effect of the length of the substituents linear aliphatic group (AG) on the physical properties (thermal properties) of the HOPCLOH and, eventually, in their poly(ester-urethanes) (PEUs) the effect on thermal and mechanical properties. Typically, the ROP of the CL was achieved in the presence of the catalyst mentioned previously and 1,4-butanediol, and after 90 minutes at 150 °C, a high conversion (98%) was detected by ^1^H NMR (Table 2). The CL/HO–(CH_2_)_*m*_–OH feed of molar ratio allowed control of degree polymerization on the HOPCLOH. By means of three analytical techniques such as NMR, GPC, and in some cases MALDI-TOF, the experimental values of *M*_n_ were acquired, showing an expected difference between *M*_*n*_s values due to the different analytical techniques, as shown in Fig. 1. *M*_n_ (NMR) values are approximating to *M*_n_ (calcd), and *M*_n_ (NMR) values were preferably used in our calculations [second step, synthesis of PEUs] because the samples have a moderate polydispersity (not narrow), and all of them are oligomers (oligoesters) with relatively high contribution by their end groups (detected by ^1^H NMR). NMR, MALDI-TOF, and GPC; overall, these results followed the next pattern: *M*_n_ (NMR) < *M*_n_ (calcd) < *M*_n_ (MALDI) < *M*_n_ (GPC). The overestimation of *M*_n_ determined by GPC for the HOPCLOHs was attributable to the polystyrene standards used in the calibration curve. Moderate polydispersity and a unimodal distribution were observed in all GPC chromatograms (*M*_w_/*M*_n_ = 1.18–1.39) (Table S3†).

**Fig. 2:**
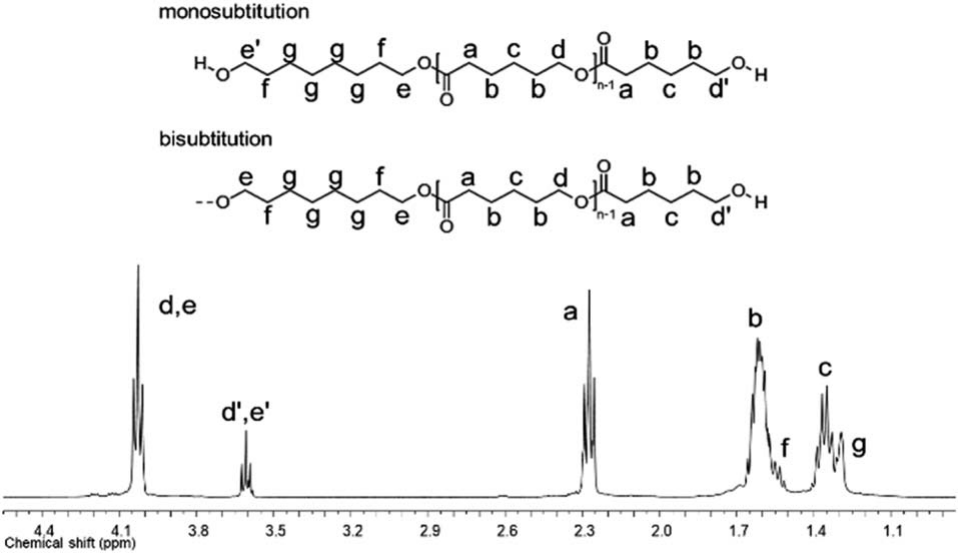
^1^H NMR (400 MHz) spectrum in CDCl_3_ at room temperature for HOPCL_8a10_OH.

**Fig. 3:**
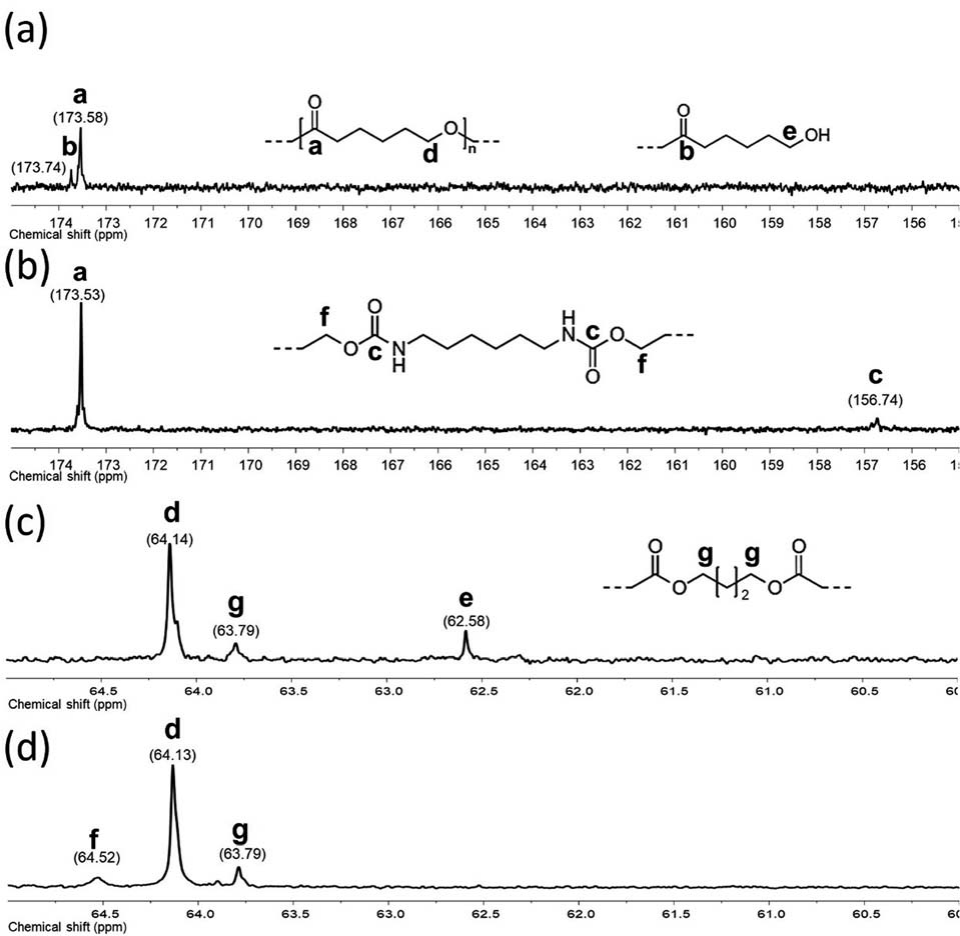
^13^C NMR (100 MHz) spectra in CDCl_3_ for: HOPCL_4a10_OH (a and c) and for PEU_4a10_A (b and d) at room temperature.

By NMR, the chemical essence of HOPCLOH samples was determined; for example, in Fig. 2, the ^1^H NMR spectrum of HOPCL_8a10_OH is shown, in which signals at 4.04 and 3.62 ppm were assigned to methylenes of the main chain of PCL [d, e, CH_2_–O–] and methylenes of the hydroxyl end group [d′, e′, CH_2_–OH][9], respectively. In the PCL, the methylene group (–CH_2_–CH_2_–) had an insertion as a monosubstitution [e′ and e, HO–CH_2(e′)_–(CH_2_)_6_–CH_2(e)_–O, *δ* 3.62 (e′) and 4.04 (e)] or bisubstitution [e, O–CH_2(e)_–(CH_2_)_6_–CH_2(e)_–O, *δ* 4.04]. In the ^13^C NMR spectrum for HOPCL_4a10_OH [Fig. 3(a and c)], a series of peaks for carbonyl (ester group) (173.66 ppm), methylenes of the main chain (64.25 ppm), and methylene attached to terminal hydroxyl groups (62.69 ppm) were detected, confirming an α,ω-hydroxy telechelic PCL.^12^ Previous reports corroborate the peak assignments for HOPCLOH in the ^1^H and ^13^C NMR spectra.^9,12,43^

**Fig. 4:**
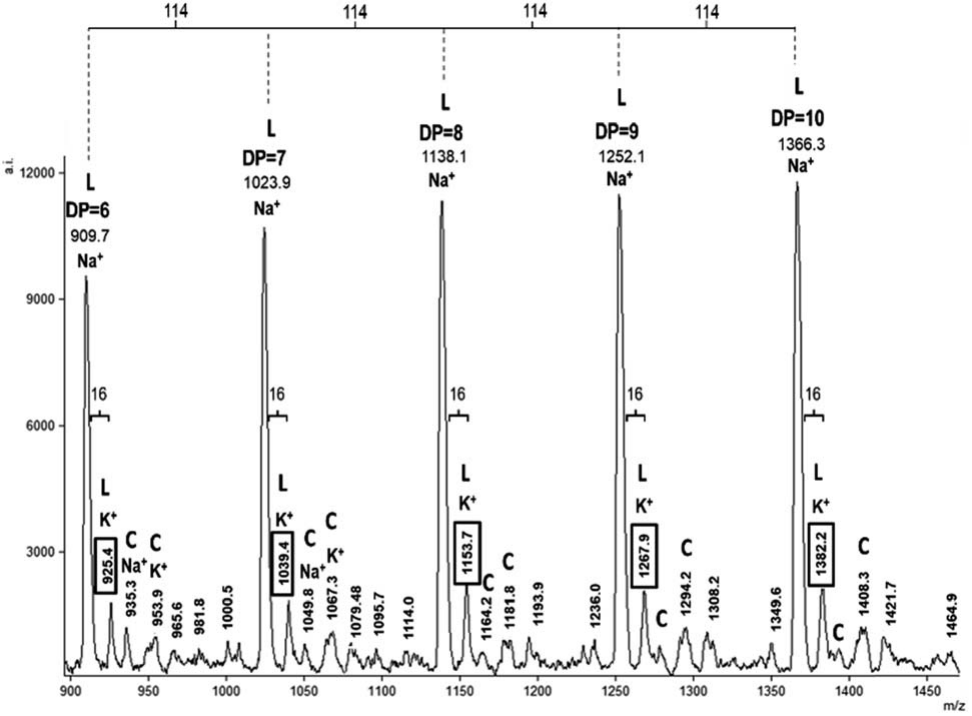
MALDI-TOF spectrum (linear mode) expanded view for the 890–1500 *m*/*z* fragments of HOPCL_12a10_OH (note: 114 and 16 are the values of the molecular weight of CL and the difference between Na^+^ and K^+^ (doping the same species of polymer), respectively).

Fig. 4 shows the MALDI-TOF spectrum of HOPCLOH using diol 1,12-dodecanodiol as initiator; the zone corresponded to fragments with 6–10 CL repeat units (Na^+^ and K^+^ ions), showing a characteristic pattern of a unimodal distribution of a HOPCLOH oligoester with a systematic increase in the DP. The most intense peaks were due to HOPCLOH (linear, L) fragments doped with Na^+^; the K^+^ doped peaks were the next in intensity. Peaks with low intensity were attributable to macrocyclic species of ε-caprolactone (CL)_*n*_ (cyclic, C). It is known that (CL)_*n*_ is a product of intramolecular transesterification.^44^ The peaks with very low intensity were ascribed to species such as α-hydroxyl-ω-(carboxylic acid) poly(ε-caprolactone) and α-hydroxyl-ω-(sodium carboxylate) PCL, the carboxylic acid group (–CO_2_H) end groups were related to traces of water as an initiator in the ROP of CL, and the carboxylate group (–CO_2_–) was produced *in situ* during the MALDI-TOF experiment.^43^ The MALDI-TOF spectra for the HOPCLOH samples using 1,4-butanediol or 1,8-octanediol as initiator, showed similar patterns (Fig. S4 and S5 of the ESI File†).

#### Effect of the degree of polymerization (DP) on α,ω-hydroxy telechelic poly(ε-caprolactone) (HOPCLOH)

The degree of polymerization (DP) of the α,ω-hydroxy telechelic poly(ε-caprolactone)s (HOPCLOHs) was a function of the CL/HO–(CH_2_)_*m*_–OH feed molar ratio, to observe the effect of the DP on the HOPCLOH, three DPs were studied (DP = 5, 10, and 15).Thermal properties of HOPCLOH were analyzed by Differential Scanning Calorimetry (DSC) (Table 3), where a systematic increase in the DP (DP = 5, 10, and 15) of each family of oligoesters (HOPCL_4_OH), (HOPCL_8_OH), and (HOPCL_12_OH) is visualized.

**Table 3:** Thermal properties of poly(ε-caprolactone) diols (HOPCLOH) with different types of linear aliphatic diols [HO(CH_2_)_*m*_OH, where *m* = 4, 8, and 12] as initiators in the ROP of CL.^a^^,^^b^ The effect of the degree polymerization (DP) on the thermal properties

Sample	Initiator	DP _(NMR)_^c^	Alkyl c^,^^d^(%)	*T*_c_ e(°C)	*T*_m_ e(°C)	Δ*H*_m_^e^(J g^−1^)	Δ*H*_mPCL_^f^(J g^−1^)	*x*_i_ g(%)
HOPCL_4a5_OH	HO(CH_2_)_4_OH	4.9	16	4	17	66	66	49
HOPCL_4a10_OH	HO(CH_2_)_4_OH	10.0	7	19	34	72	72	53
HOPCL_4a15_OH	HO(CH_2_)_4_OH	14.7	5	24	39	79	79	58
HOPCL_8a5_OH	HO(CH_2_)_8_OH	5.0	23	9	19	80	62	46
HOPCL_8a10_OH	HO(CH_2_)_8_OH	10.1	11	19	33	73	65	48
HOPCL_8a15_OH	HO(CH_2_)_8_OH	14.0	9	22	39	79	72	53
HOPCL_12a5_OH	HO(CH_2_)_12_OH	5.0	29	14	23	105	74	54
HOPCL_12a10_OH	HO(CH_2_)_12_OH	10.1	15	24	35	93	79	58
HOPCL_12a15_OH	HO(CH_2_)_12_OH	15.0	11	28	41	88	78	58

a Effect of the number of methylenes in HOPCLOH. HOPCLOH,α,ω-hydroxy telechelic poly(ε-caprolactone); ROP, ring-opening polymerization; CL, ε-caprolactone; PCL, poly(ε-caprolactone); DSC, differential scanning calorimetry.

b HOPCLOH = HO–PCL–OH or HO–(CH_2_)_*m*_–PCL–OH, where *m* = 4, 8, and 12.

c Determined by ^1^H NMR in CDCl_3_.

d Calculated from the equation alkyl (%) = (MW_initiator_/*M*_n_ (NMR)) × 100, where MW_initiator_ is the molecular weight of initiator or alkyl diol (HOROH).

e Obtained by DSC analysis.

f Calculated from the equation Δ*H*_mPCL_ = Δ*H*_m_ − (Δ*H*_m_·*x*_alkyl_) where *x*_alkyl_ is the weight fraction of alkyl group in the HOCLOH oligoester.

g Quantified from Δ*H*_m_.

**Fig. 5:**
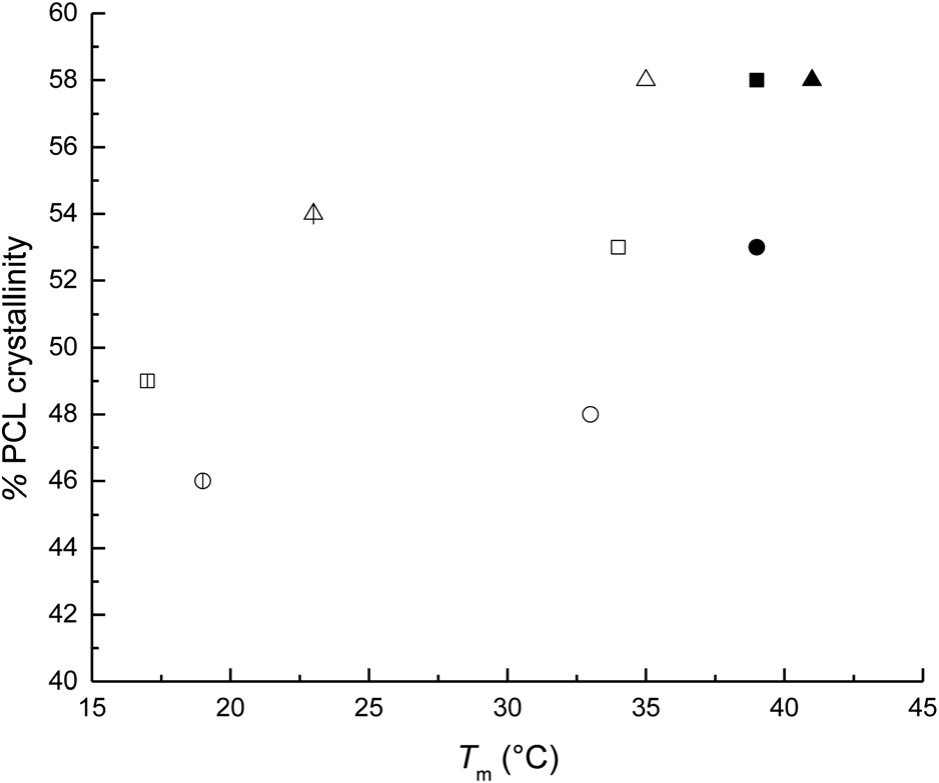
Dependence of melting point (*T*_m_) and crystallinity (*x*_i_) in macrodiols (HOPCLOH). The macrodiols with HO(CH_2_)_4_OH as initiator are indicated with squares (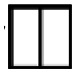 DP = 5; □ DP = 10, and ■ DP = 15); macrodiols with HO(CH_2_)_8_OH as initiator are indicated with circles (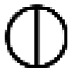 DP = 5, ○ DP = 10, and ● DP = 15) and macrodiols with HO(CH_2_)_12_OH as initiator are indicated with triangles (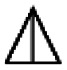 DP = 5, △ DP = 10, and ▲ DP = 15).

**Fig. 6:**
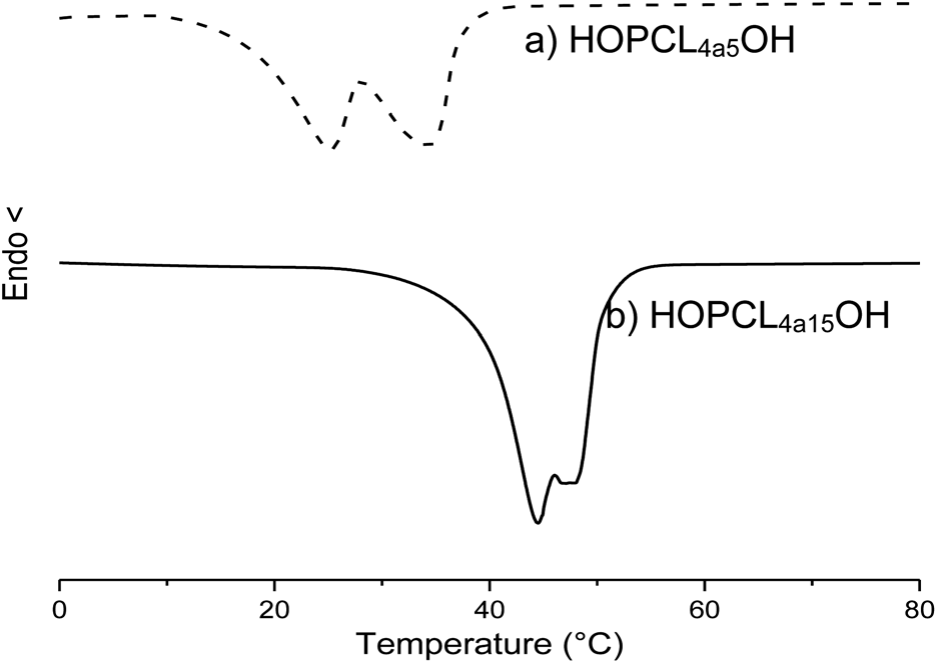
DSC thermograms of macrodiols with HO(CH_2_)_4_OH as initiator and (a) DP = 5 (HOPCL_4a5_OH), and (b) DP = 15 (HOPCL_4a15_OH), showing a double peak of melting temperature (*T*_m_) Table 3.

The weight percent of the alkyl group was decreased from HOPCL_4a5_OH (16%) to HOPCL_4a15_OH (5%), and it had an inversely proportional effect on the DP; this effect was similarly repeated in the rest of HOPCLOHs. The values of melting temperatures (*T*_m_) and crystallinity (*x*_i_) increased proportionally to the DP (Fig. 5); this was because the crystalline microdomains of the PCL increased when the main chain of the oligoester was longer, favoring the lamellar thickness. The melting temperature (*T*_m_) exhibited a pronounced double peak for HOPCLOH samples with DP = 5 (Fig. 6), this profile could be understood as two different sizes of crystallites in two different environments, some found in a more amorphous region (which will have a slightly lower *T*_m_) and others in more crystalline areas (which will have a higher *T*_m_). Additionally, this effect of the double peak in the *T*_m_ of HOPCLOH samples with DP = 5 could be attributed to a disruption of the crystal domains of PCL at low DP and the effect of the monosubstitution fraction visualized by NMR.

#### Impact of the number of methylenes of the initiator [(CH_2_)_*m*_] on α,ω-hydroxy telechelic poly(ε-caprolactone) (HOPCLOH)

**Fig. 7:**
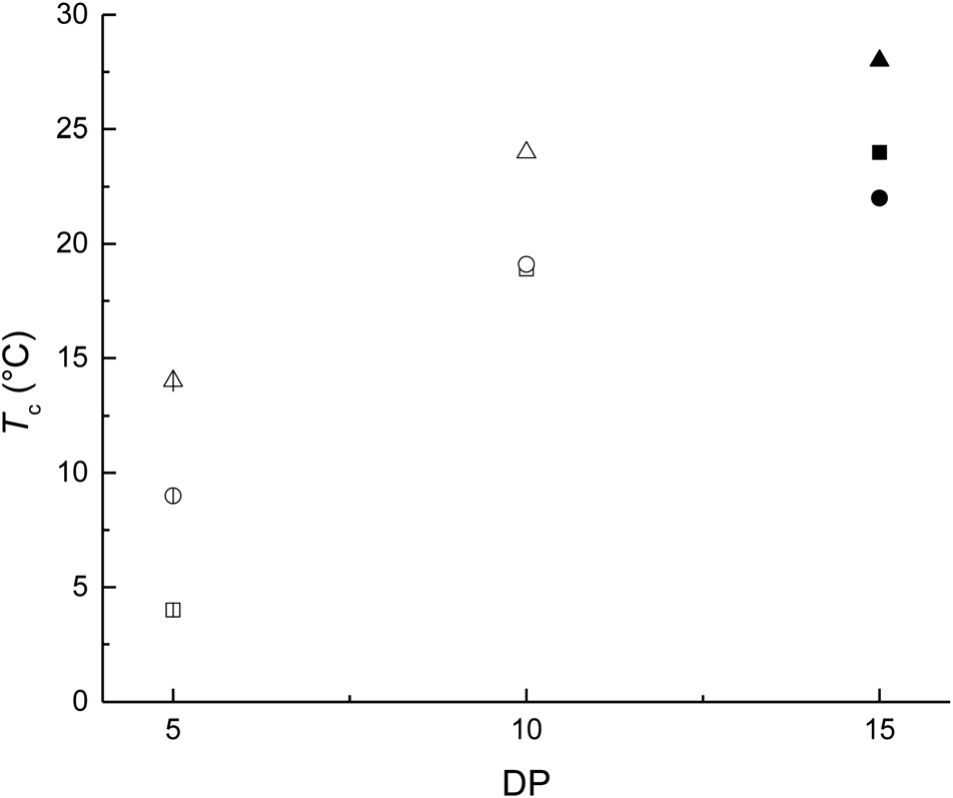
Dependence of crystallization temperature (*T*_c_) and degree of polymerization (DP) in macrodiols (HOPCLOH). The macrodiols with HO(CH_2_)_4_OH as initiator are indicated with squares (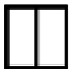 DP = 5; □ DP = 10, and ■ DP = 15); macrodiols with HO(CH_2_)_8_OH as initiator are indicated with circles (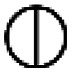 DP = 5, ○ DP = 10, and ● DP = 15) and macrodiols with HO(CH_2_)_12_OH as initiator are indicated with triangles (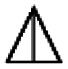 DP = 5, △ DP = 10, and ▲ DP = 15).

All samples were synthesized with similar number-average molecular weight (*M*_n_) taking in consideration the degree of polymerization (*M*_n_) (*M*_n_(calcd) = 600–800 Da, DP = 5; *M*_n_ (calcd) = 1200–1400 Da, DP = 10; *M*_n_ (calcd) = 1600–1800 Da, DP = 15). This allowed us to compare the effect of methylene (CH_2_)_*m*_ groups of three different types of linear aliphatic diols with a systematic increase in the number of methylenes [HO(CH_2_)_*m*_OH, where *m* = 4, 8, and 12] used as initiators to synthesize the HOPCLOHs. For example, in the case of a DP = 5, the weight percent of the alkyl group increased when the number of methylenes [(CH_2_)_*m*_] changed from 4 to 12, from HOPCL_4a5_OH (16%) to HOPCL_12a5_OH (29%), evidencing the effect of the length of the weight percentage of the alkyl group in the oligoester, since when the methylene chain increased, the crystallization temperature (*T*_c_) and melting temperature (*T*_m_) increased proportionally, from HOPCL_4a5_OH (*T*_c_ = 4 °C; *T*_m_ = 17 °C) to HOPCL_12a5_OH (*T*_c_ = 14 °C; *T*_m_ = 23 °C). The number of methylenes in the initiator (CH_2_)_*m*_ influenced the *T*_c_, where –(CH_2_)_12_– induced a relative high value in the series in the HOPCLOH oligoester species, which can be appreciated in Fig. 7. This effect was attributed to the crystalline domain of the 12-methylene segment inducing a nucleation effect. Complementarily, the number of methylenes (initiator) inserted in the main chain of the polyester also had a significant effect on the crystallinity (*x*_i_) of HOPCLOH, in the case of DP = 5 from 49 [(CH_2_)_4_] to 54% [(CH_2_)_12_], this is consistent with the profile of *T*_m_. So, a long aliphatic chain of methylenes can induce an increase in the crystallinity of PCL.

### Part 2. Poly(ester-urethanes) (PEUs)

**Fig. 8:**
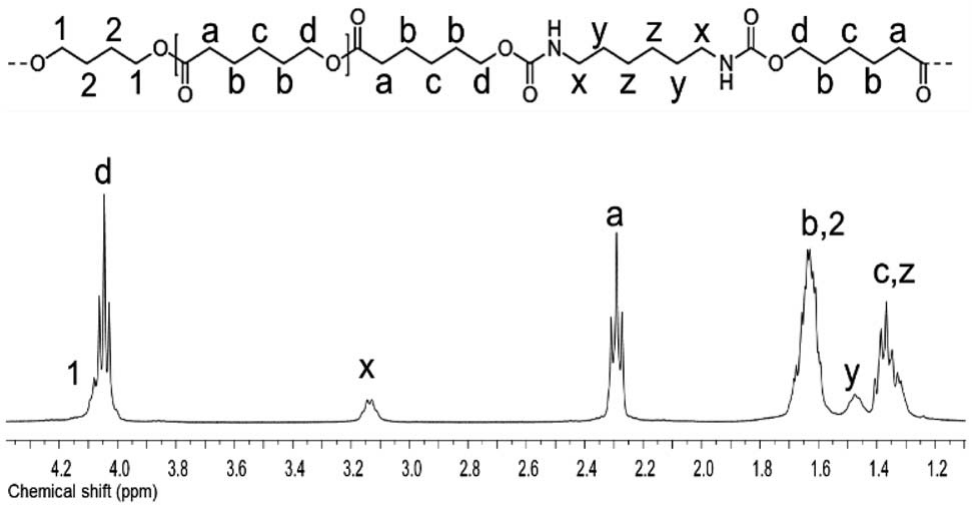
^1^H NMR (400 MHz) spectrum in CDCl_3_ at room temperature for PEU_4a10_A.

PEUs were synthesized using the HOPCLOH samples and three different diisocyanates (1,6-hexamethylene diisocyanate (HDI), 4,4′-methylene dicyclohexyl diisocyanate (HMDI), and methylene diphenyl diisocyanate (MDI)) (Scheme 1b). In a typical reaction, HOPCL_4a10_OH and diisocyanate (HDI, HMDI, or MDI) reacted with a molar ratio of 1 : 1.1, respectively, in the presence of tin(ii) 2-ethylhexanoate [Sn(Oct)_2_] as a catalyst dissolved in 1,2-dichloroethane (DCE) solvent at 80 °C for 3 h. Afterwards, a film was obtained by casting at room temperature to allow slow solvent evaporation for 12 h. This methodology was used to prepare all twenty-seven PEUs samples, varying the number of methylenes [(–CH_2_–)_*m*_, where *m* = 4, 8, and 12] of the aliphatic group in the HOPCLOH, the degree of polymerization, and the type of diisocyanate used (Table S3†). Fig. 8 shows the ^1^H NMR spectrum of PEU_4a10_A, signals at 3.13 and 1.48 ppm were assigned to methylenes of the urethane chain [*y*, –O–(C=O)–HN–CH_2(*x*)_–CH_2(*y*)_–], respectively. Also, signals at 4.05 and 2.29 ppm were assigned to methylenes of the PCL main chain [d, CH_2_–O–], and methylenes next to the carbonyl [a, CH_2_–C=O], respectively. To corroborate the chemical essence of PEUs, ^13^C NMR spectra for PEU_4a10_A, PEU_4a10_B, and PEU_4a10_C were obtained (Fig. S1–S3†), confirming the functionality of the PEUs. Comparing the ^13^C NMR spectrum of the oligomer HOPCL_4a10_OH (Fig. 3(a)) with one of their PEU with HDI as diisocyanate, PEU_4a10_A [Fig. 3(b)], carbonyl peaks at 173.65 (a) and 156.86 (c) ppm attributed to ester and urethane groups, respectively, confirmed the functionality of the PEUs. In the PEU_4a10_A precursor [Fig. 3(c)] there was the typical peak of methylene attached to a hydroxyl group (e, –CH_2_–OH), which was absent in the PEU_4a10_A [Fig. 3(d)] due to its reaction with the diisocyanate to produce urethane groups (f, –CH_2_–O–CO–NH–).

**Fig. 9:**
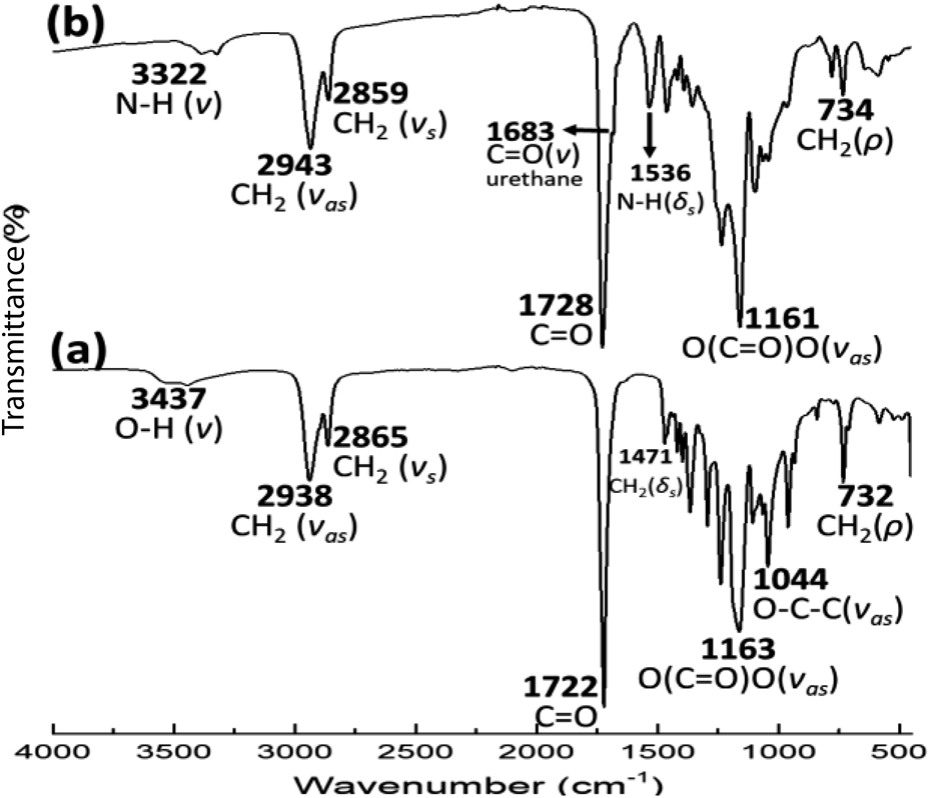
FT-IR spectra of (a) HOPCL_8a10_OH and (b) PEU_8a10_A.

The FTIR spectrum for PEU_8a10_A [Fig. 9(b)] presented distinctive bands at 3322, 1683, and 1536 cm^−1^ ascribed to vibrations of the urethane group (–O–C=O–NH) with N–H (*ν*, stretching) C=O (*ν*, stretching) and N–H (*δ*, bending), respectively. Also, a band assigned [Fig. 9(a)] to the carbonyl of the ester group in PCL (1721–1723 cm^−1^, *ν*, stretching) was identified. In the twenty-seven PEU samples, signals of unreacted diisocyanate (HDI, MDI or HMDI) (∼2270–2250 cm^−1^) were not observed, confirming the formation of urethane groups.

#### Influence of the degree of polymerization (DP) and the number of methylenes of the initiator of HOPCLOH on PEUs

**Fig. 10:**
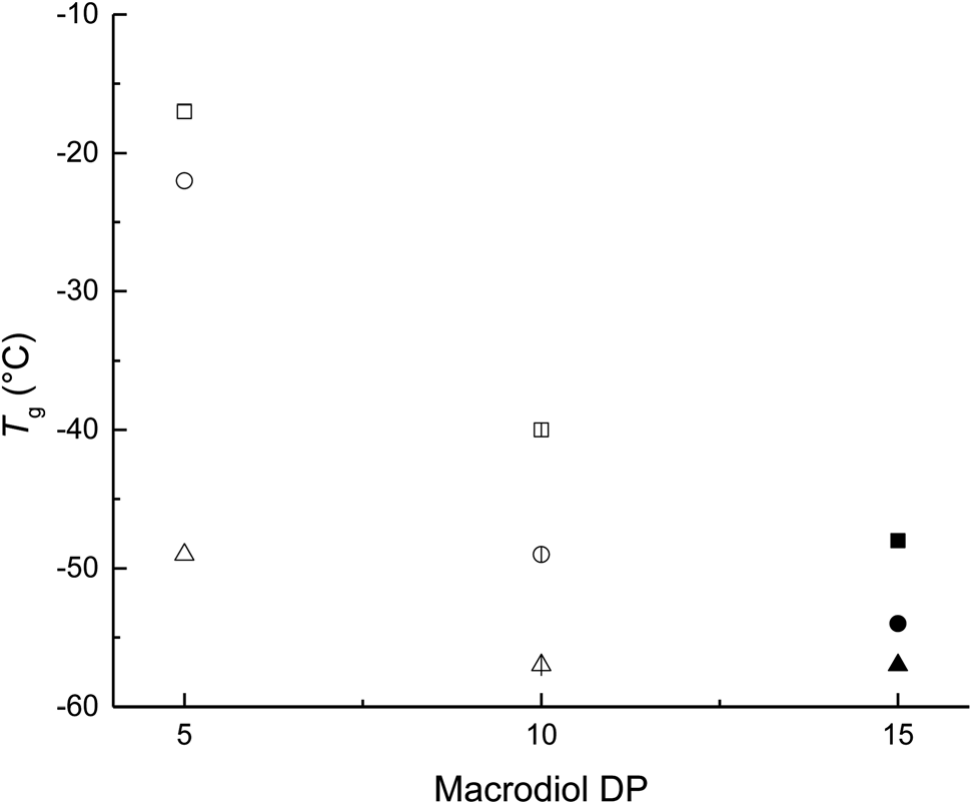
Effect of the degree of polymerization (DP) of the macrodiol on the glass transition temperature (*T*_g_) in poly(ester-urethanes) (PEUs) derived from poly(ε-caprolactone) diol (HOPCLOH) with HO(CH_2_)_4_OH as initiator and three different diisocyanates (1,6-hexamethylene diisocyanate (HDI) indicated with triangles, methylene diphenyl diisocyanate (MDI) indicated with squares, and 4,4′-methylenebis (cyclohexyl isocyanate) (HMDI) indicated with circles). The PEUs derived from the macrodiol HOPCL_4a_OH with DP = 5 are indicated with open figures □, △, and ○ ; PEUs derived from macrodiols with DP = 10 are indicated with lined figures 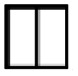, 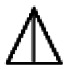, and 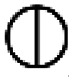; and PEUs derived from macrodiols with DP = 15 are indicated with filled figures ■, ▲, and ●.

The effects of different degrees of polymerization (DP) in the HOPCLOH used for the synthesis of PEU were analyzed regarding their thermal properties (physical properties) using scanning differential calorimetry (DSC). DSC analysis for all PEU samples with HDI and PEUs derived from HOPCL_4_OH with MDI and HMDI is presented in Table 4. The comparison of HOPCLOH with respect to PEUs demonstrated a decrease in the enthalpy of fusion (Δ*H*_m_) and crystallinity (*x*_i_) of PCL microdomains, following the same pattern as the HOPCLOH samples, that is, by increasing the DP in each HOPCLOH with different aliphatic diols, Δ*H*_m_ and *x*_i_ increased. The decrease in *x*_i_ of PCL was largely attributed to the presence of physical cross-links of the urethane groups in the main chain of PEU.^35^ PEU samples synthesized with HDI presented two different transitions: (1) glass transition temperature (*T*_g_) and (2) melting temperature (*T*_m_). Fig. 10 shows the influence of the DP of HOPCLOH macrodiols on the *T*_g_ of PEUs, showing that the DP had a significant effect, where the *T*_g_ decreased inversely proportional to the DP, according to the repetitive units in PCL increase. This involves: (1) more flexibility attributed to the methylenes [–CO(CH_2_)_5_O–] in the main chain of PCL, which produced a PEU with a lower *T*_g_ from DP = 5 to 15; and (2) the hydrogen bonding interactions induced by urethane groups were reduced by the steric hindrance of long PCL chains (DP 15). Usually, the classic value of *T*_g_ for the PCL is −60 °C; therefore, all PEUs in Fig. 10 have high *T*_g_ values with respect to the regular PCL homopolymer, indicating that PEUs are in principle a more rigid material than PCL. The pattern of *T*_g_ of the PEUs with respect to the diisocyanate used as a precursor is MDI (aromatic) > HMDI (cyclic aliphatic) > HDI (linear aliphatic). So, MDI produced a PEU that was more rigid due to the aryl groups; on the other hand, HDI produced a PEU that was more flexible due to the six linear methylenes.

**Table 4:** Thermal properties of poly(ester-urethanes) (PEUs) derived from poly(ε-caprolactone) diols (HOPCLOH) and three different types diisocyanates (1,6-hexamethylene diisocyanate (HDI), methylene diphenyl diisocyanate (MDI), and 4,4′-methylenebis (cyclohexyl isocyanate) (HMDI)). Effect of the number of methylenes in HOPCLOH on the PEU^a^,^b^

Sample	Precursor	HS c^,^^e^(%)	SS d^,^^e^(%)	*T*_g_ f(°C)	*T*_c_ f(°C)	*T*_m_ f(°C)	Δ*H*_m_^f^(J g^−1^)	Δ*H*_mPCL_^g^(J g^−1^)	*χ*_i_ h(%)	Alkyl i(%)
PEU_4a5_A	HOPCL_4a5_OH	24	76	−49	—	51	10	10	7	16
PEU_4a10_A	HOPCL_4a10_OH	14	86	−57	—	31	15	15	11	8
PEU_4a15_A	HOPCL_4a15_OH	10	90	−57	—	43	29	29	22	5
PEU_8a5_A	HOPCL_8a5_OH	23	77	−49	—	51	13	10	7	23
PEU_8a10_A	HOPCL_8a10_OH	13	87	−58	—	30	19	16	12.5	12
PEU_8a15_A	HOPCL_8a15_OH	10	90	−61	—	41	31	28	20	9
PEU_12a5_A	HOPCL_12a5_OH	22	78	−47	—	58	15	12	9	29
PEU_12a10_A	HOPCL_12a10_OH	13	87	−58	—	36	19	16	12	16
PEU_12a15_A	HOPCL_12a15_OH	10	90	−58	—	46	29	26	19	11
PEU_4a5_B	HOPCL_4a5_OH	33	67	−17	—	—	—	—	—	16
PEU_4a10_B	HOPCL_4a10_OH	20	80	−40	—	—	—	—	—	8
PEU_4a15_B	HOPCL_4a15_OH	14	86	−48	−3	35	25	25	18	5
PEU_4a5_C	HOPCL_4a5_OH	33	67	−22	—	—	—	—	—	16
PEU_4a10_C	HOPCL_4a10_OH	21	79	−49	—	—	—	—	—	8
PEU_4a15_C	HOPCL_4a15_OH	15	85	−54	3	37	20	20	15	5

a HOPCLOH,α,ω-hydroxy telechelic poly(ε-caprolactone); PCL, poly(ε-caprolactone); PEU, poly(ester-urethane); HS, hard segment; SS, soft segment; A, 1,6-hexamethylene diisocyaanate (HDI); B, methylene diphenyl diisocyanate (MDI); C, 4,4′-methylenebis (cyclohexyl isocyanate) (HMDI). DSC, differential scanning calorimetry.

b HOPCLOH = HO–PCL–OH or HO–(CH_2_)_*m*_–PCL–OH, where *m* = 4, 8, and 12.

c Hard segment.

d Soft segment.

e Weight percent.

f Obtained by DSC analysis.

g Calculated from the equation Δ*H*_mPCL_ = Δ*H*_m_**·***x*_ss_ where *x*_ss_ is the weight fraction of soft segment in the HOCLOH oligoester.

h Quantified from Δ*H*_m_.

i Calculated with respect to the HOPCLOH precursor. Percent determined by ^1^H NMR in CDCl_3_. Calculated from the equation alkyl (%) = (MW_initiator_/*M*_n_(NMR)) × 100, where MW_initiator_ is the molecular weight of initiator or alkyl diol (HOROH).

The *T*_m_ in PEUs is mainly attributed to the crystal domain of PCL soft segments, in the case of PEU_10_A derived from HDI and HOPCLOH exhibited a decrease of *T*_m_ respect to PEU_5_A and PEU_15_A. This phenome can be explained in terms of DP, where DP = 10 of HOPCLOH after its reaction induces a dispersion of the lamella crystalline domains of PCL which is embedded into the amorphous domain of PCL. In contrast, the shorter chains in PEU5A and the longer chains in PEU15A induce bigger crystallites of lamella crystalline domains favoring a relative high *T*_m_ respect to PEU_10_A. This explanation is according to the values of enthalpy and crystallinity of all PEU series, where the crystallinity of PEU is proportional to the DP of HOPCLOH as precursor.

On half of the PEUs synthetized with MDI and HMDI, a fusion was not observed, indicating that those samples were amorphous. So, a diisocyanate with a bulky substituent (MDI and HMDI) produced a urethane group that induced a disorder on the PCL chains, generating an amorphous domain. In the case of PEUs with DP = 15, except on PEUs with HDI, a third transition is observed: (3) crystallization temperature (*T*_c_) (*T*_c_ = −31 to 3 °C); the existence of these three transitions is similar to the reported for the PLLA oligomers of moderate molecular weight (2100–3700 Da).^45–47^ Therefore, the DP in oligomers from HOPCLOH was a crucial factor in inducing transitions such as *T*_g_, *T*_c_, and *T*_m_. Mostly amorphous PEUs were obtained at low DP of PCL in the case of non-linear diisocyanates.

**Fig. 11:**
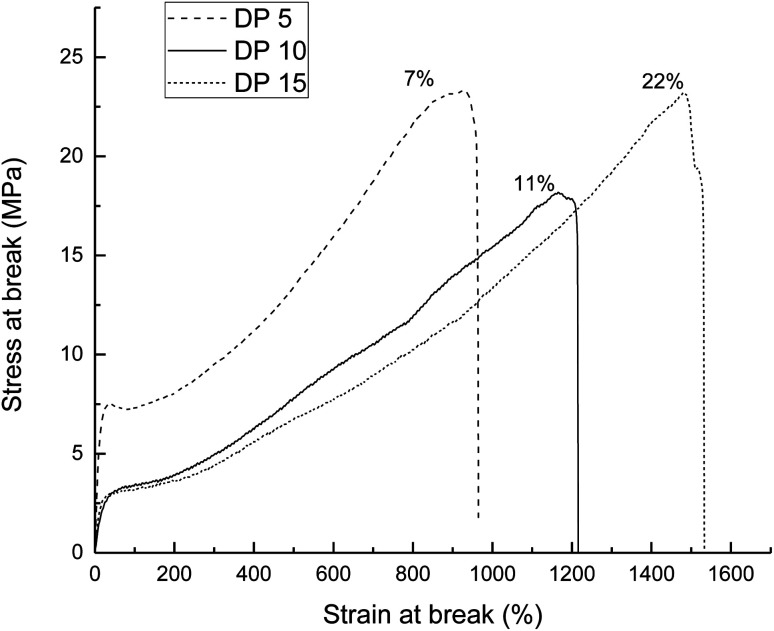
Stress–strain curves for PEUs derived from poly(ε-caprolactone) diol (HOPCLOH) with HO(CH_2_)_4_OH as an initiator using HDI and with different DP. The numbers next to each graph indicate the % PCL crystallinity.

For the mechanical properties of PEUs, for example, PEU derived from HOPCLOH with HO(CH_2_)_4_OH as an initiator, with HDI and DP = 5 showed a high modulus value (54.5 MPa), which was attributed to the high content of hard segments and low crystallinity (*x*_i_ = 7%) of PCL. Additionally, the crystallinity of the three PEUs (from DP 5 to 15) contributed to the strain at break in a proportional manner (Fig. 11). Complementarily, for PEUs, prepared using HOPCLOHs with HO(CH_2_)_4_OH as an initiator and with DP = 15 (using MDI or HMDI), the value of modulus increased, which is consistent with a significant value of *x*_i_ (15–18%). In general, a plastic behavior was observed for the majority of the PEUs samples.

#### Effect of the type of diisocyanate used for synthesis of PEUs

**Fig. 12:**
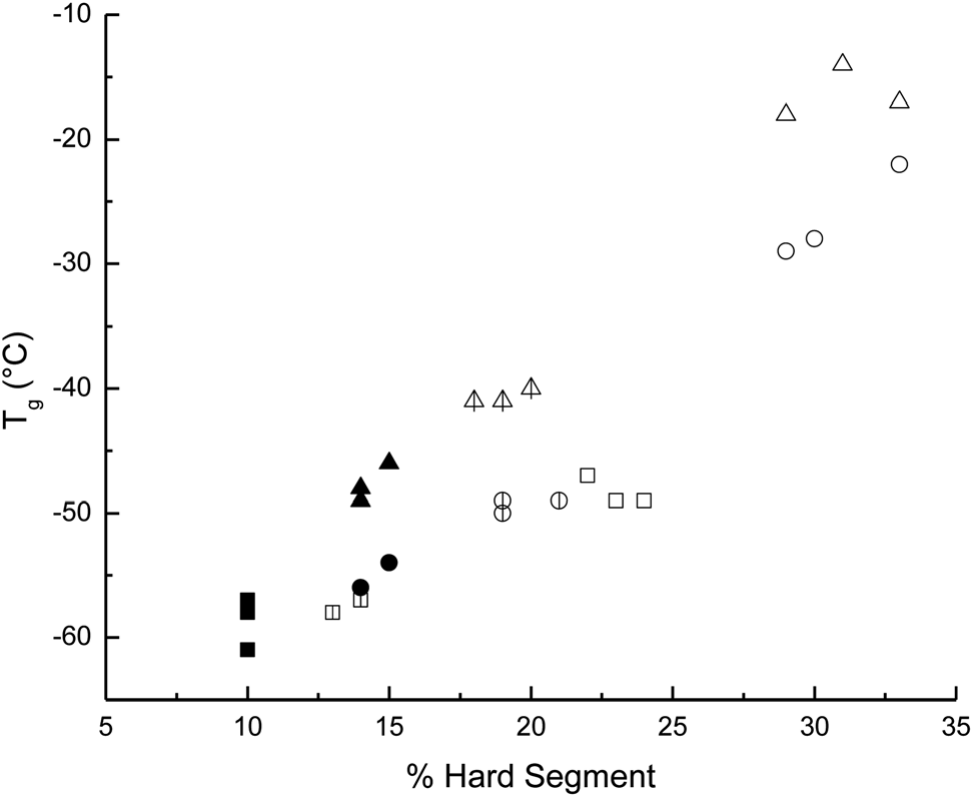
Effect of hard segment content on the glass transition temperature (*T*_g_) in poly(ester-urethanes) (PEUs) derived from poly(ε-caprolactone) diols (HOPCLOH) and three different diisocyanates (1,6-hexamethylene diisocyanate (HDI) indicated with squares, methylene diphenyl diisocyanate (MDI) indicated with triangles, and 4,4′-methylenebis (cyclohexyl isocyanate) (HMDI) indicated with circles). The PEUs derived from the macrodiols with DP = 5 are indicated with open figures □, △, and ○ PEUs derived from macrodiols with DP = 10 are indicated with lined figures 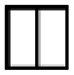, 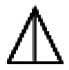, and 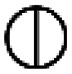; and PEUs derived from macrodiols with DP = 15 are indicated with filled figures ■, ▲, and ●.

The main effect of the increase in the content of diisocyanate was the decrease in the enthalpy of fusion and crystallinity of PCL microdomains in PEUs with respect to HOPCLOH because the urethane groups caused a disruption in the order of the main chains of PCL and favored the hard segment interactions (Table S5†). As the percentage of hard segments increased, the *T*_g_ increased (Fig. 12). The rise in the hard segment contributed to greater stiffness in the polyurethane due to physical crosslinking (hydrogen bonding) between the urethane and carbonyl groups. Comparing the types of diisocyanate, it was evident that the PEU derived from MDI (PEU_4a5_B, *T*_g_ = −17 °C) exhibited the highest and the HDI (PEU_4a5_A, *T*_g_ = −49 °C) the lowest values of *T*_g_ (Table 4); this phenomenon was explained in terms of the aromatic (MDI) *vs.* aliphatic (HDI) hard segment, where the flexibility of aliphatic (methylene groups) substituents induced the lowest values of *T*_g_. On the contrary, the rigidity of aromatic (aryl group) substituents produced high values of *T*_g_.

**Fig. 13:**
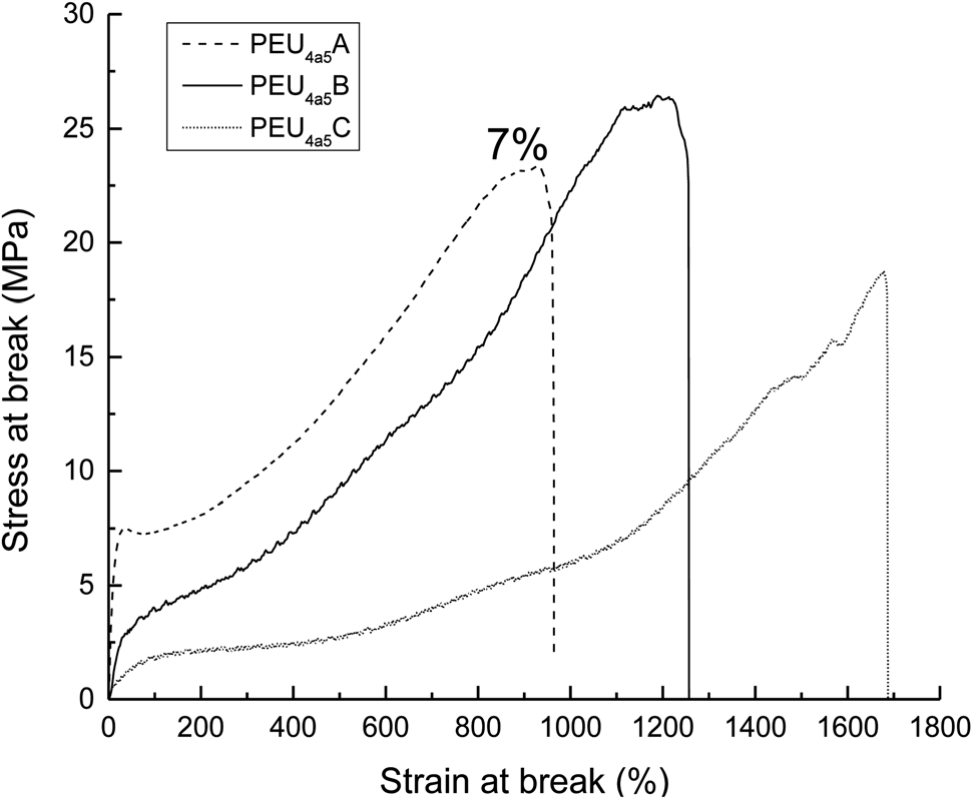
Stress–strain curves for PEUs derived from poly(ε-caprolactone) diol (HOPCLOH) with HO(CH_2_)_4_OH as initiator and DP of 5. The number next to PEU_4a5_A graph indicate the % PCL crystallinity, the other two PEUs does not present crystallinity. Where the end letter of the name of the sample corresponds to; (A) 1,6-hexamethylene diisocyanate (HDI); (B) methylene diphenyl diisocyanate (MDI); (C) 4,4′-methylenebis (cyclohexyl isocyanate) (HMDI).

The Young modulus, tensile strength, and elongation at break derived from stress–strain curves are summarized in Table S5.† The modulus was affected by the type of diisocyanate; when using linear diisocyanate (HDI) with DP = 5, the modulus was higher than when using bulkier diisocyanates such as MDI and HMDI, the latter having slightly lower modulus values than PEUs with MDI, as shown in Fig. 13. So, the volume of the substituents (aromatic and cyclic) in the hard segment (urethane group) induced a disorder on the crystallinity domain of PCL, generating an amorphous domain and decreasing the modulus, which was consistent with comparisons made between PEUs with HDI and PEUs with MDI.^24^ Complementarily, the PEUs derived from HDI (DP = 5, Table S5,† PEU_4a5_A, PEU_8a5_A, and PEU_12a5_A) formed urethane groups where the intermolecular hydrogen bonding was favorable because the six methylenes (in HDI) were not bulky groups; this was the reason for the increase in the modulus. In general, the results of stress at break do not have a pattern. However, the profile of PEUs showed mainly plastic behavior.

This work contributes to the synthesis and characterization of poly(ester-urethanes) (PEUs) derived from PCL. Other studies, such as types of solvents in the synthesis of PEUs and degradation behavior, are currently underway in our laboratory.

## Conclusions

Nine different macrodiols derived from α,ω-hydroxy telechelic poly(ε-caprolactone) (HOPCLOH) were synthesized by ROP of ε-caprolactone using a series of linear aliphatic diols [HO(CH_2_)_*m*_OH, where *m* = 4, 8, and 12] as initiators and three different degrees of polymerization (DP) of 5, 10, and 15, to explore the effect of initiator and DP, resulting in a controlled polymerization. In HOPCLOH, the values of crystallinity (*x*_i_) increased proportionally to the degree of polymerization (DP). Also, the melting temperature (*T*_m_) clearly exhibited a proportional dependency on the DP. Complementarily, a longer alkyl group such as twelve methylenes (CH_2_)_12_ inserted in the main chain of HOPCLOH favored a relatively high *x*_i,_ and crystallization temperature (*T*_c_) was attributed to the nucleation effect. Twenty-seven poly(ester-urethanes) (PEUs) derived from HOPCLOH and three different diisocyanates [1,6-hexamethylene diisocyanate (HDI), 4,4′-methylene dicyclohexyl diisocyanate (HMDI), and methylene diphenyl diisocyanate (MDI)] were prepared. The thermal properties of PEUs showed a dependency on the type of diisocyanate, where linear HDI induced high values of *x*_i_ with respect to MDI and HMDI. This effect was attributed to the disruption of the PCL crystalline domains due to the bulky substituents (MDI and HMDI). The glass transition temperature (*T*_g_) of PEUs depended mainly on the percent of the hard segment (HS%) (or content of diisocyanates), where a high value of HS% produced a high *T*_g_ for all series. Complementarily, the DP in HOPCLOH oligomers was a significant factor in inducing the *T*_m_ of PEUs. Mostly amorphous PEUs were obtained at low DP (DP = 5 and 10) in the case of diisocyanates such as MDI and HMDI, which were not linear. In terms of the effects that affect the crystallinity for (1) HOPCLOH and (2) PEUs, these were: (1) DP > initiator, and (2) diisocyanate > DP > initiator, respectively. The mechanical properties indicated that factors such as HS% and *x*_i_ had a significant contribution to the modulus of PEU. Most of the PEU samples exhibited plastic behavior.

## Data availability

The data supporting this article have been included as part of the ESI.†

## Author contributions

Miriam Paola Barrera-Nava: investigation, validation, formal analysis, writing – original draft. Rodrigo Navarro: investigation, supervision. Ángel Marcos-Fernández: investigation, supervision. José E. Báez: conceptualization, supervision, writing – original draft, writing – review, funding acquisition.

## Conflicts of interest

There are no conflicts to declare.

## Supplementary Material

RA-014-D4RA03951C-s001
