# Supplementary material for: Synthesis and characterization of macrodiols and non-segmented poly(ester-urethanes) (PEUs) derived from α,ω-hydroxy telechelic poly(ε-caprolactone) (HOPCLOH): effect of initiator, degree of polymerization, and diisocyanate
Source: RSC Adv. 2024 Aug 27;14(37):27241–51. doi: 10.1039/d4ra03951c (PMC11348852; doi:10.1039/d4ra03951c)
Supplement: RA-014-D4RA03951C-s001 [file RA-014-D4RA03951C-s001.pdf]

Supplementary Information (SI) for RSC Advances.
This journal is © The Royal Society of Chemistry 2024

Supplementary Information

Synthesis and characterization of macrodiols and non-segmented poly(ester-urethanes) derived from α,ω-hydroxy telechelic poly(ε-caprolactone) (HOPCLOH). Effect of initiator, degree of polymerization, and diisocyanate

Miriam P. Barrera-Nava[1], Rodrigo Navarro[2], Ángel Marcos-Fernández[2], José E. Báez[1]*

[1] Department of Chemistry, University of Guanajuato (UG), Noria Alta S/N, 36050 Guanajuato, Gto, Mexico.

[2] Institute of Polymer Science and Technology, CSIC, C/Juan de la Cierva No. 3, 28006 Madrid, Spain.

Correspondence to: José E. Báez. Email: jebaez@ugto.mx

Table of contents

Tables		Page
	**Table S1.** Poly(ε-caprolactone) diols (HOPCLOH) prepared using different types of linear aliphatic diols [$HO(CH_2)_mOH$, where m=4, 8, and 12] as initiators in the ROP of CL.[a]	4
	**Table S2.** Poly(ester-urethanes) (PEUs) derived from poly(ε-caprolactone) diols (HOPCLOH) and three different types diisocyanates (HDI, MDI, and HMDI).	5
	**Table S3.** Number-average molecular weight obtained by two different techniques of the HOPCLOH prepared using different types of linear aliphatic diols [$HO(CH_2)_mOH$, where m=4, 8, and 12] as initiators in the ROP of CL.[a]	6
	**Table S4.** Complete thermal properties of poly(ester-urethanes) (PEUs) derived from poly(ε-caprolactone) diols (HOPCLOH) and three different types diisocyanates (HDI, MDI, and HMDI). Effect of the number of methylenes in HOPCLOH on the PEU.[a]	7
	**Table S5.** Mechanical properties of poly(ester-urethanes) (PEUs) derived from poly(ε-caprolactone) diols (HOPCLOH) and three different diisocyanates (HDI, MDI, and HMDI). Effect of the number of methylenes in HOPCLOH on the PEU.[a]	9
**Figures**		
	**Fig. S1.** ^{13}C NMR (100 MHz) spectrum in $CDCl_3$ at room temperature for $PEU_{4a10}A$.	10
	**Fig. S2.** ^{13}C NMR (100 MHz) spectrum in $CDCl_3$ at room temperature for $PEU_{4a10}B$.	11
	**Fig. S3.** ^{13}C NMR (100 MHz) spectrum in $CDCl_3$ at room temperature for $PEU_{4a10}C$.	12
	**Fig. S4.** MALDI-TOF spectrum (linear mode) expanded view for the 1000−2000 m/z fragments of $HOPCL_{4a10}OH$ (note: 114 and 16 are the values of the molecular weight of CL and the difference between Na^+ and K^+ (doping the same species of polymer), respectively).	13
	**Fig. S5.** MALDI-TOF spectrum (linear mode) expanded view for the 1000−2000 m/z fragments of $HOPCL_{8a10}OH$ (note: 114 and 16 are the values of the molecular weight of CL and the difference between Na^+ and K^+ (doping the same species of polymer), respectively).	14
	**Fig. S6.** Effect of number of methylenes on the crystallinity PCL for poly(ester-urethanes) (PEUs) derived from poly(ε-caprolactone) diols (HOPCLOH) and 1,6-hexamethylene diisocyanate (HDI). The PEUs derived from the macrodiols with DP=5 as initiator are indicated with open squares □, and macrodiols are indicated with	15

filled square ■ ; PEUs derived from macrodiols with DP=10 as initiator are indicated with open circles ○, and macrodiols are indicated with filled circles ●, and PEUs derived from macrodiols with DP=15 as initiator are indicated with open triangles △, and macrodiols are indicated with filled triangle▲.

Fig. S7. Effect of Hard Segment (HS) content on the crystallinity PCL for poly(ester-urethanes) (PEUs) derived from poly(ε-caprolactone) diols (HOPCLOH) and 1,6-hexamethylene diisocyanate (HDI). The PEUs derived from the macrodiols with $HO(CH_2)_4OH$ as initiator are indicated with squares (◫ DP=5; □ DP=10, and ■ 16
DP=15); PEUs derived from macrodiols with $HO(CH_2)_8OH$ as initiator are indicated with circles (⦶ DP=5, ○ DP=10, and ●DP=15) and PEUs derived from macrodiols with $HO(CH_2)_{12}OH$ as initiator are indicated with triangles (⚠ DP=5, △ DP=10, and ▲ DP=15).

Fig. S8. Mechanical properties of the synthesized PEUs with HDI: a) stress at break, b) strain at break, and c) modulus. The PEUs derived from the macrodiols with $HO(CH_2)_4OH$ as initiator are indicated with squares (◫
DP=5; □ DP=10, and ■ DP=15); PEUs derived from 17
macrodiols with $HO(CH_2)_8OH$ used as initiator are indicated with circles (⦶ DP=5, ○ DP=10, and ● DP=15) and PEUs derived from macrodiols with $HO(CH_2)_{12}OH$ as initiator are indicated with triangles (⚠ DP=5, △ DP=10, and ▲ DP=15).

Table S1. Poly(ε-caprolactone) diols (HOPCLOH) prepared using different types of linear aliphatic diols [$HO(CH_2)_mOH$, where m=4, 8, and 12] as initiators in the ROP of CL.[a]

DP (cald)	Initiator		
	1,4-BD[b]	1,8-OD[c]	1,12-DD[d]
5	$HOPCL_{4a5}OH$	$HOPCL_{8a5}OH$	$HOPCL_{12a5}OH$
10	$HOPCL_{4a10}OH$	$HOPCL_{8a10}OH$	$HOPCL_{12a10}OH$
15	$HOPCL_{4a15}OH$	$HOPCL_{8a15}OH$	$HOPCL_{12a15}OH$

[a]Polymerization at 150°C for 90 min with 65 mmol of CL, three CL/initiator molar ratios of 5,10, and 15 were used in the experiments ($DP_{(calcd)}$=5, 10, or 15) and ammonium decamolybdate as a catalyst. HOPCLOH= HO ─ PCL ─ OH or HO ─ $(CH_2)_m$ ─ PCL─ OH, where m = 4, 8, and 12.

[b]1,4-Butanediol

[c]1,8-Octanediol

[d]1,12-Dodecanediol

Table S2. Poly(ester-urethanes) (PEUs) derived from poly(ε-caprolactone) diols (HOPCLOH) and three different types diisocyanates (HDI, MDI, and HMDI).

**Diisocyanate**	**1,4-BD**		
	HOPCL$_{4a5}$OH	HOPCL$_{4a10}$OH	HOPCL$_{4a15}$OH
HDI	PEU$_{4a5}$A	PEU$_{4a10}$A	PEU$_{4a15}$A
MDI	PEU$_{4a5}$B	PEU$_{4a10}$B	PEU$_{4a15}$B
HMDI	PEU$_{4a5}$C	PEU$_{4a10}$C	PEU$_{4a15}$C
**Diisocyanate**	**1,8-OD**		
	HOPCL$_{8a5}$OH	HOPCL$_{8a10}$OH	HOPCL$_{8a15}$OH
HDI	PEU$_{8a5}$A	PEU$_{8a10}$A	PEU$_{8a15}$A
MDI	PEU$_{8a5}$B	PEU$_{8a10}$B	PEU$_{8a15}$B
HMDI	PEU$_{8a5}$C	PEU$_{8a10}$C	PEU$_{8a15}$C
**Diisocyanate**	**1,12-DD**		
	HOPCL$_{12a5}$OH	HOPCL$_{12a5}$OH	HOPCL$_{12a5}$OH
HDI	PEU$_{12a5}$A	PEU$_{12a10}$A	PEU$_{12a15}$A
MDI	PEU$_{12a5}$B	PEU$_{12a10}$B	PEU$_{12a15}$B
HMDI	PEU$_{12a5}$C	PEU$_{12a10}$C	PEU$_{12a15}$C

Table S3. Number-average molecular weight obtained by two different techniques of the HOPCLOH prepared using different types of linear aliphatic diols [$HO(CH_2)_mOH$, where m=4, 8, and 12] as initiators in the ROP of CL.[a]

Sample	Initiator	Alkyl(%)[b,c]	Conv(%)[b]	DP(calcd)	M_n(calcd)[d]	M_n(NMR)[d,e]	M_n(SEC)[f]	M_w/M_n[f]	Ratio[g]
$HOPCL_{4a5}OH$	$HO(CH_2)_4OH$	16	98	5	650	570	1868	1.23	0.38
$HOPCL_{4a10}OH$	$HO(CH_2)_4OH$	8	98	10	1230	1120	2575	1.33	0.48
$HOPCL_{4a15}OH$	$HO(CH_2)_4OH$	5	99	15	1760	1660	3300	1.37	0.53
$HOPCL_{8a5}OH$	$HO(CH_2)_8OH$	23	99	5	710	640	1819	1.19	0.39
$HOPCL_{8a10}OH$	$HO(CH_2)_8OH$	12	99	10	1300	1210	2585	1.33	0.50
$HOPCL_{8a15}OH$	$HO(CH_2)_8OH$	9	99	15	1750	1630	3184	1.36	0.55
$HOPCL_{12a5}OH$	$HO(CH_2)_{12}OH$	29	99	5	770	690	1849	1.18	0.41
$HOPCL_{12a10}OH$	$HO(CH_2)_{12}OH$	16	99	10	1350	1250	2651	1.32	0.51
$HOPCL_{12a15}OH$	$HO(CH_2)_{12}OH$	11	99	15	1910	1780	3716	1.39	0.51

[a]Polymerization at 150°C for 90 min with 65 mmol of CL, three CL/initiator molar ratios of 5,10, and 15 were used in the experiments ($DP_{(calcd)}$=5, 10, or 15) and ammonium decamolybdate as a catalyst. HOPCLOH= HO ─ PCL ─ OH or HO ─ $(CH_2)_m$ ─ PCL─ OH, where m = 4, 8, and 12.

[b]Determined by ^1H NMR in $CDCl_3$.

[c]Obtained from the equation alkyl (%) = ($MW_{initiator}/M_n$(NMR)) x 100, where $MW_{initiator}$ is the molecular weight of initiator or alkyl diol (HOROH).

[d]Obtained from the equation M_n(calcd) = (MW(CL))(mmol CL/mmol ROH) + MW(HOROH), where MW is the molecular weight of ε-caprolatone monomer or aliphatic diol (HOROH).

[e]Obtained from the equation M_n(NMR) = ($DP_{(PCL)}$ x MW(CL)) + MW(ROH), where MW is the molecular weight of ε-caprolactone monomer or aliphatic diol (HOROH).

[f]Determined by SEC results.

[g]M_n(calcd)/M_n(SEC) ratio.

Table S4. Complete thermal properties of poly(ester-urethanes) (PEUs) derived from poly(ε-caprolactone) diols (HOPCLOH) and three different types diisocyanates (HDI, MDI, and HMDI). Effect of the number of methylenes in HOPCLOH on the PEU.[a]

Sample	Precursor	HS (%)[b,d]	SS (%)[b,d]	T_g (°C)[e]	T_c (°C)[e]	T_m (°C)[e]	ΔH_m (J/g)[e]	ΔH_{mPCL} (J/g)[f]	χ_i (%)[g]	Alkyl (%)[h]
$PEU_{4a5}A$	$HOPCL_{4a5}OH$	24	76	-49	-	51	10	10	7	16
$PEU_{4a10}A$	$HOPCL_{4a10}OH$	14	86	-57	-	31	15	15	11	8
$PEU_{4a15}A$	$HOPCL_{4a15}OH$	10	90	-57	-	43	29	29	22	5
$PEU_{8a5}A$	$HOPCL_{8a5}OH$	23	77	-49	-	51	13	10	7	23
$PEU_{8a10}A$	$HOPCL_{8a10}OH$	13	87	-58	-	30	19	16	12.5	12
$PEU_{8a15}A$	$HOPCL_{8a15}OH$	10	90	-61	-	41	31	28	20	9
$PEU_{12a5}A$	$HOPCL_{12a5}OH$	22	78	-47	-	58	15	12	9	29
$PEU_{12a10}A$	$HOPCL_{12a10}OH$	13	87	-58	-	36	19	16	12	16
$PEU_{12a15}A$	$HOPCL_{12a15}OH$	10	90	-58	-	46	29	26	19	11
$PEU_{4a5}B$	$HOPCL_{4a5}OH$	33	67	-17	-	-	-	-	-	16
$PEU_{4a10}B$	$HOPCL_{4a10}OH$	20	80	-40	-	-	-	-	-	8
$PEU_{4a15}B$	$HOPCL_{4a15}OH$	14	86	-48	-3	35	25	25	18	5
$PEU_{8a5}B$	$HOPCL_{8a5}OH$	31	69	-14	-	-	-	-	-	23
$PEU_{8a10}B$	$HOPCL_{8a10}OH$	19	81	-41	-	-	-	-	-	12
$PEU_{8a15}B$	$HOPCL_{8a15}OH$	15	85	-46	-1	29	13	11	8	9
$PEU_{12a5}B$	$HOPCL_{12a5}OH$	29	71	-18	-	-	-	-	-	29
$PEU_{12a10}B$	$HOPCL_{12a10}OH$	18	82	-41	-	13	1	1	1	16
$PEU_{12a15}B$	$HOPCL_{12a15}OH$	14	86	-49	-12	33	24	21	16	11
$PEU_{4a5}C$	$HOPCL_{4a5}OH$	33	67	-22	-	-	-	-	-	16
$PEU_{4a10}C$	$HOPCL_{4a10}OH$	21	79	-49	-	-	-	-	-	8
$PEU_{4a15}C$	$HOPCL_{4a15}OH$	15	85	-54	3	37	20	20	15	5
$PEU_{8a5}C$	$HOPCL_{8a5}OH$	30	70	-28	-	35	1	1	1	23
$PEU_{8a10}C$	$HOPCL_{8a10}OH$	19	81	-50	-	-	-	-	-	12
$PEU_{8a15}C$	$HOPCL_{8a15}OH$	15	85	-54	2	31	6	5	4	9
$PEU_{12a5}C$	$HOPCL_{12a5}OH$	29	71	-29	-	-	-	-	-	29
$PEU_{12a10}C$	$HOPCL_{12a10}OH$	19	81	-49	-	21	2	2	1	16
$PEU_{12a15}C$	$HOPCL_{12a15}OH$	14	86	-56	-31	34	27	24	18	11

HOPCLOH,α,ω-hydroxy telechelic poly(ε-caprolactone); PCL, poly(ε-caprolactone); PEU, poly(ester-urethane); HS, hard segment; SS, soft segment; A, 1,6-hexamethylene diisocyaanate (HDI); B, methylene diphenyl diisocyanate (MDI); C, 4,4′-methylenebis (cyclohexyl isocyanate) (HMDI)). DSC, differential scanning calorimetry.

[a]HOPCLOH= HO – PCL – OH or HO – $(CH_2)_m$ – PCL– OH, where m = 4, 8, and 12.

[b]Hard segment.

[c]Soft segment.

[d]Weight percent.

[e]Obtained by DSC analysis.

[f]Calculated from the equation $\Delta H_{mPCL} = \Delta H_m \cdot x_{ss}$) where x_{ss} is the weight fraction of soft segment in the HOCLOH oligoester.

[g]Quantified from ΔH_m.

[h]Calculated with respect to the HOPCLOH precursor. Percent determined by ^{1}H NMR in $CDCl_3$. Calculated from the equation alkyl (%) = ($MW_{initiator}/M_n$(NMR)) x 100, where $MW_{initiator}$ is the molecular weight of initiator or alkyl diol (HOROH).

Table S5. Mechanical properties of poly(ester-urethanes) (PEUs) derived from poly(ε-caprolactone) diols (HOPCLOH) and three different diisocyanates (HDI, MDI, and HMDI). Effect of the number of methylenes in HOPCLOH on the PEU.[a]

Sample	Precursor	Stress at break (MPa)	Strain at break (%)	Modulus (MPa)	χ_i (%)[b]
$PEU_{4a5}A$	$HOPCL_{4a5}OH$	23.7 ± 1.70	948 ± 98	54.5 ± 3.0	7.0
$PEU_{4a10}A$	$HOPCL_{4a10}OH$	19.1 ± 1.39	1197 ± 13	2.9 ± 1.1	11.0
$PEU_{4a15}A$	$HOPCL_{4a15}OH$	23.6 ± 3.32	1503 ± 148	5.9 ± 5.3	22.0
$PEU_{8a5}A$	$HOPCL_{8a5}OH$	31.4 ± 2.06	1399 ± 101	89.9 ± 8.2	7.0
$PEU_{8a10}A$	$HOPCL_{8a10}OH$	32.4± 4.57	1527 ± 71	5.0 ± 1.3	12.5
$PEU_{8a15}A$	$HOPCL_{8a15}OH$	26.1 ± 4.13	1641 ± 144	3.1 ± 0.6	20.0
$PEU_{12a5}A$	$HOPCL_{12a5}OH$	22.4 ± 3.35	774 ± 99	87.9 ± 13.4	9.0
$PEU_{12a10}A$	$HOPCL_{12a10}OH$	30.9 ± 6.62	1482 ± 168	5.1 ± 2.8	12.0
$PEU_{12a15}A$	$HOPCL_{12a15}OH$	19.6 ± 2.37	1198 ± 235	108.0 ± 2.0	19.0
$PEU_{4a5}B$	$HOPCL_{4a5}OH$	24.4 ± 3.85	1193 ± 96	4.1 ± 1.0	-
$PEU_{4a10}B$	$HOPCL_{4a10}OH$	19.3 ± 2.10	1747 ± 81	2.9 ± 0.5	-
$PEU_{4a15}B$	$HOPCL_{4a15}OH$	30.6 ± 4.85	1684 ± 144	63.8 ± 6.2	18.0
$PEU_{8a5}B$	$HOPCL_{8a5}OH$	16.8 ± 2.51	1177 ± 39	4.4 ± 1.5	-
$PEU_{8a10}B$	$HOPCL_{8a10}OH$	19.3 ± 3.50	1705 ± 134	4.3 ± 0.8	-
$PEU_{8a15}B$	$HOPCL_{8a15}OH$	22.1 ± 2.41	1201 ± 71	13.1 ± 10.7	8.0
$PEU_{12a5}B$	$HOPCL_{12a5}OH$	13.9 ± 2.29	1088 ± 55	3.9 ± 1.1	-
$PEU_{12a10}B$	$HOPCL_{12a10}OH$	19.3 ± 1.37	1670 ± 92	11.5 ± 18.5	1.0
$PEU_{12a15}B$	$HOPCL_{12a15}OH$	29.2 ± 4.1	1552 ± 61	43.9 ± 22.7	16.0
$PEU_{4a5}C$	$HOPCL_{4a5}OH$	22.7 ± 2.98	1678 ± 48	5.6 ± 1.8	-
$PEU_{4a10}C$	$HOPCL_{4a10}OH$	9.2 ± 1.17	1778 ± 155	1.4 ± 0.2	-
$PEU_{4a15}C$	$HOPCL_{4a15}OH$	14.4 ± 1.31	865 ± 32	95.6 ± 11.1	15.0
$PEU_{8a5}C$	$HOPCL_{8a5}OH$	11.2 ± 3.60	2023 ± 95	2.8 ± 1.0	1.0
$PEU_{8a10}C$	$HOPCL_{8a10}OH$	9.3 ± 1.48	2931 ± 229	1.3 ± 0.3	-
$PEU_{8a15}C$	$HOPCL_{8a15}OH$	39.6 ± 3.16	1710 ± 65	5.0 ± 0.6	4.0
$PEU_{12a5}C$	$HOPCL_{12a5}OH$	21.0 ± 3.53	1424 ± 61	8.4 ± 2.5	-
$PEU_{12a10}C$	$HOPCL_{12a10}OH$	13.8 ± 4.40	1571 ± 78	3.1 ± 1.2	1.0
$PEU_{12a15}C$	$HOPCL_{12a15}OH$	20.2 ± 2.23	1442 ± 211	42.5 ± 3.8	18.0

HOPCLOH, α,ω-hydroxy telechelic poly(ε-caprolactone); PCL, poly(ε-caprolactone); PEU, poly(ester-urethane); A, 1,6-hexamethylene diisocyanate (HDI); B, methylene diphenyl diisocyanate (MDI); C, 4,4′-methylenebis (cyclohexyl isocyanate) (HMDI)). DSC, differential scanning calorimetry.

[a]HOPCLOH= HO ─ PCL ─ OH or HO ─ $(CH_2)_m$ ─ PCL─ OH, where m = 4, 8, and 12.

[b]Obtained by DSC.

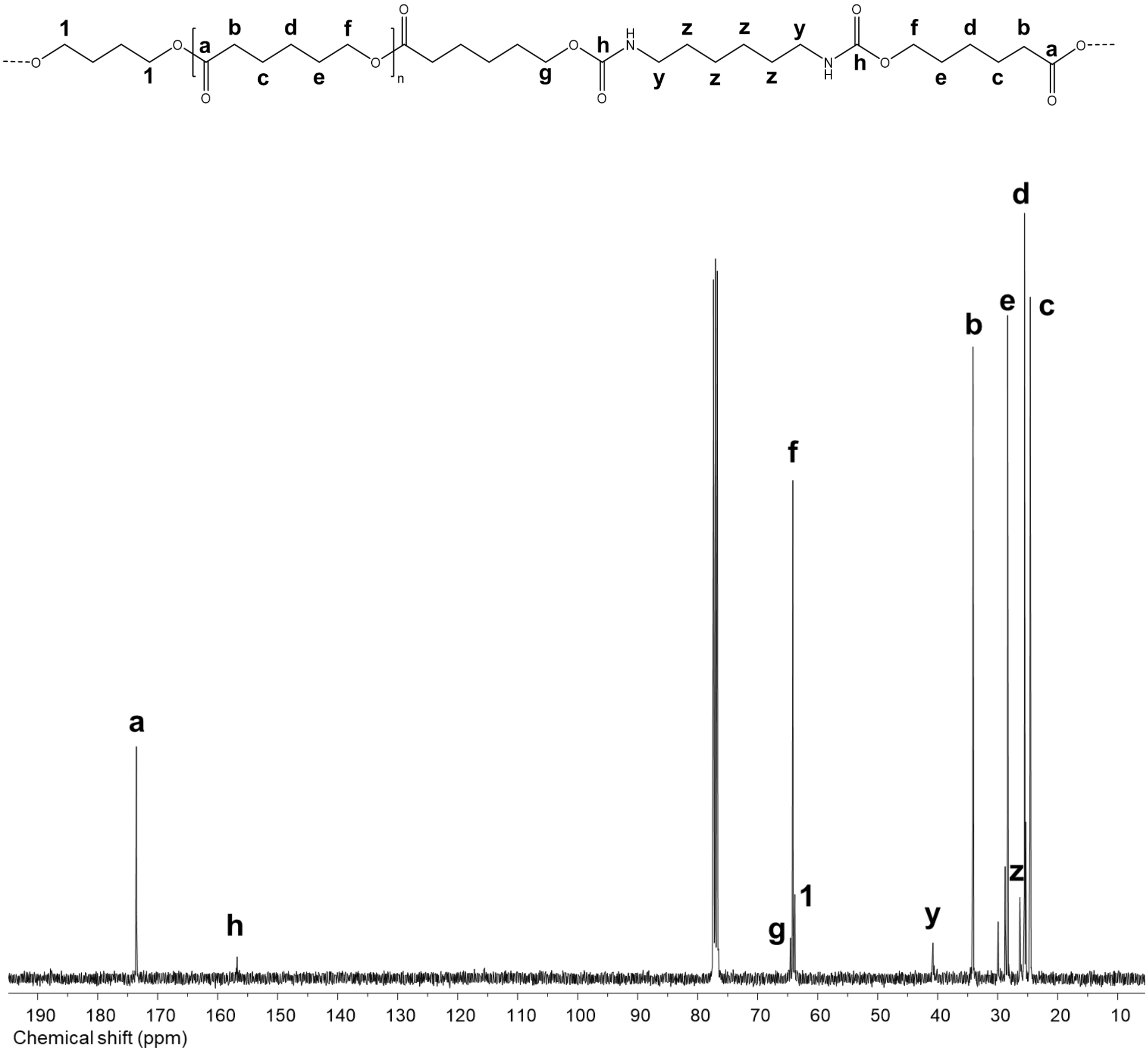

Fig. S1. ^{13}C NMR (100 MHz) spectrum in $CDCl_3$ at room temperature for $PEU_{4a10}A$.

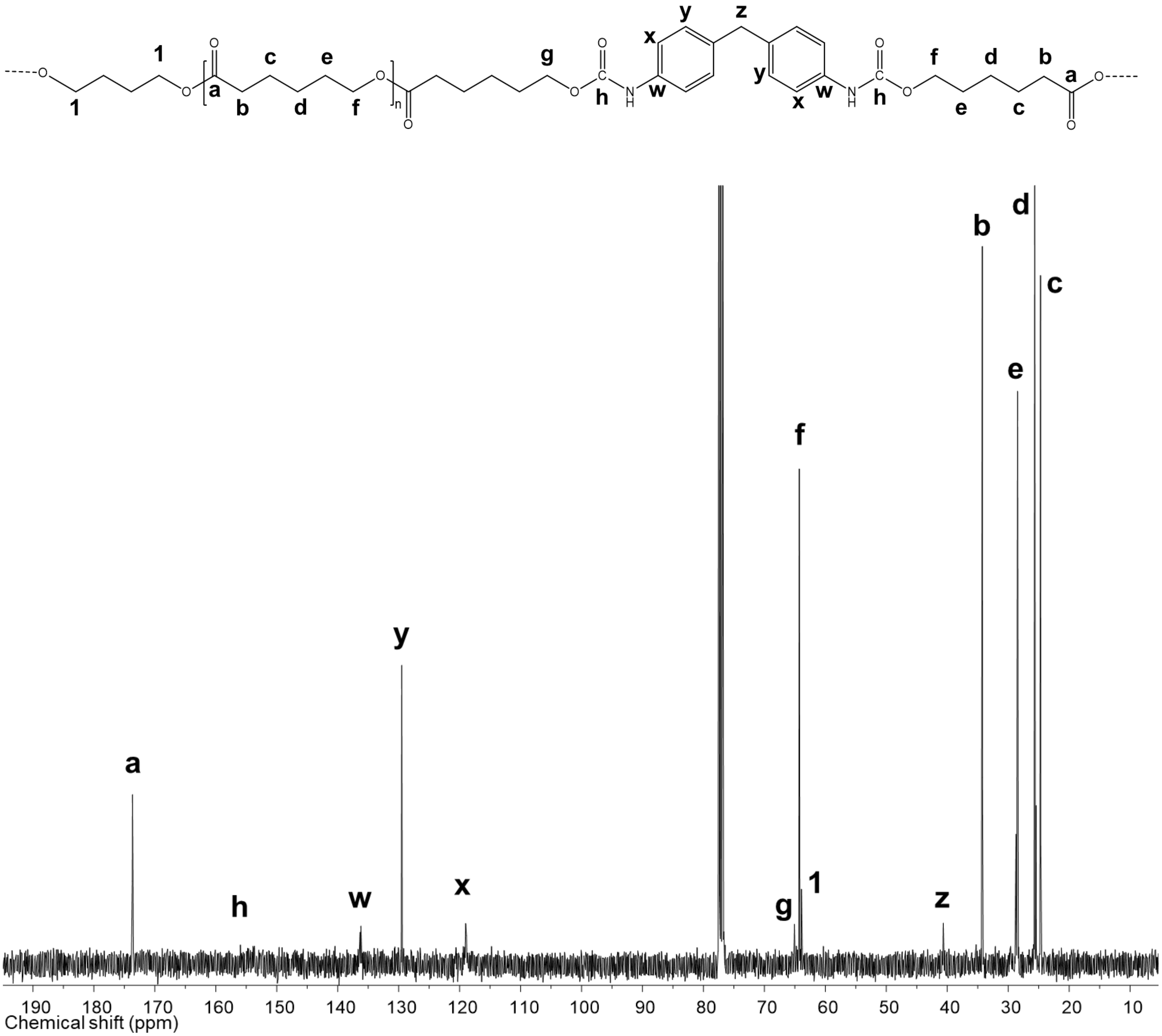

Fig. S2. ^{13}C NMR (100 MHz) spectrum in $CDCl_3$ at room temperature for $PEU_{4a10}B$.

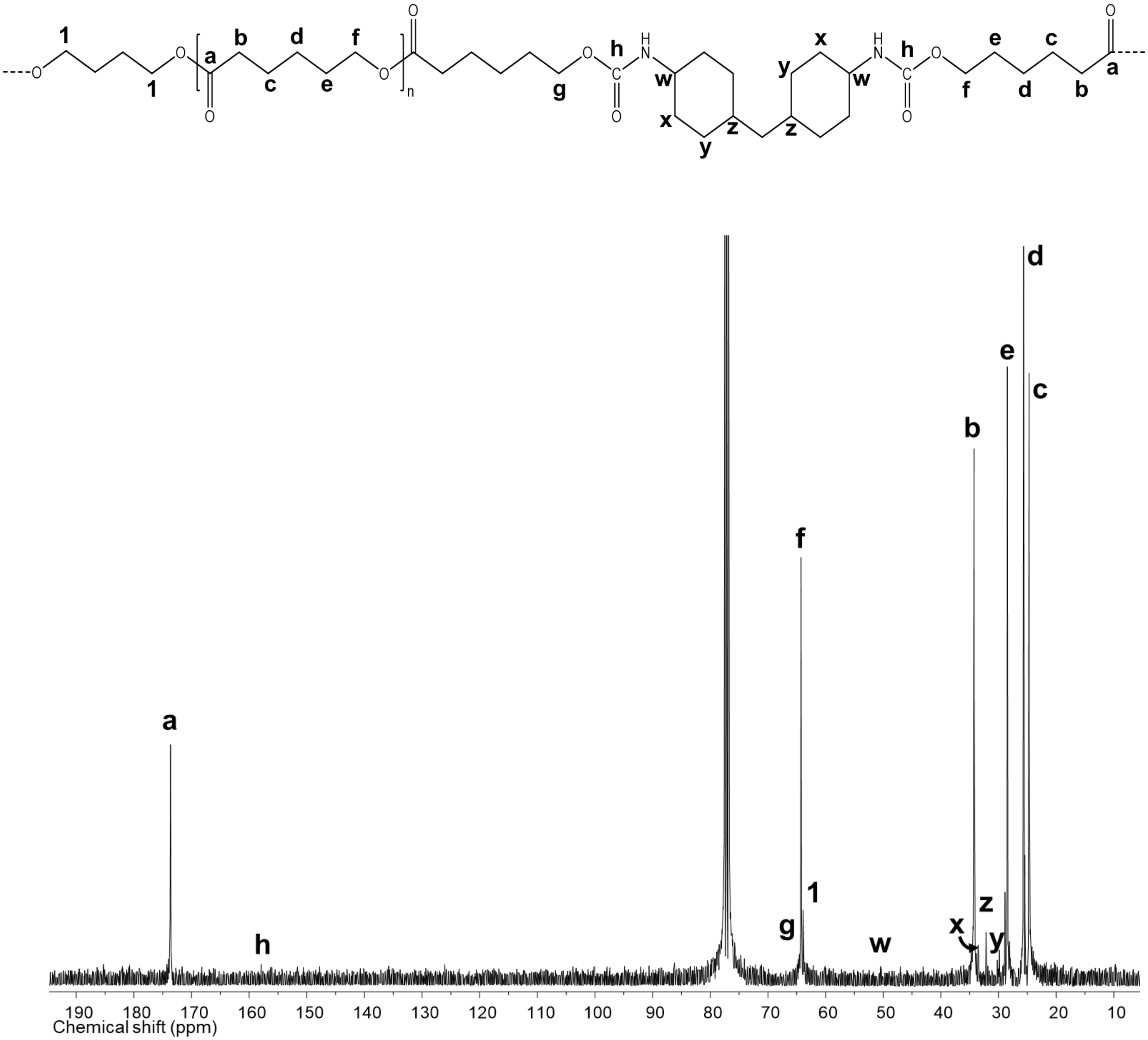

Fig. S3. ^{13}C NMR (100 MHz) spectrum in $CDCl_3$ at room temperature for $PEU_{4a10}C$.

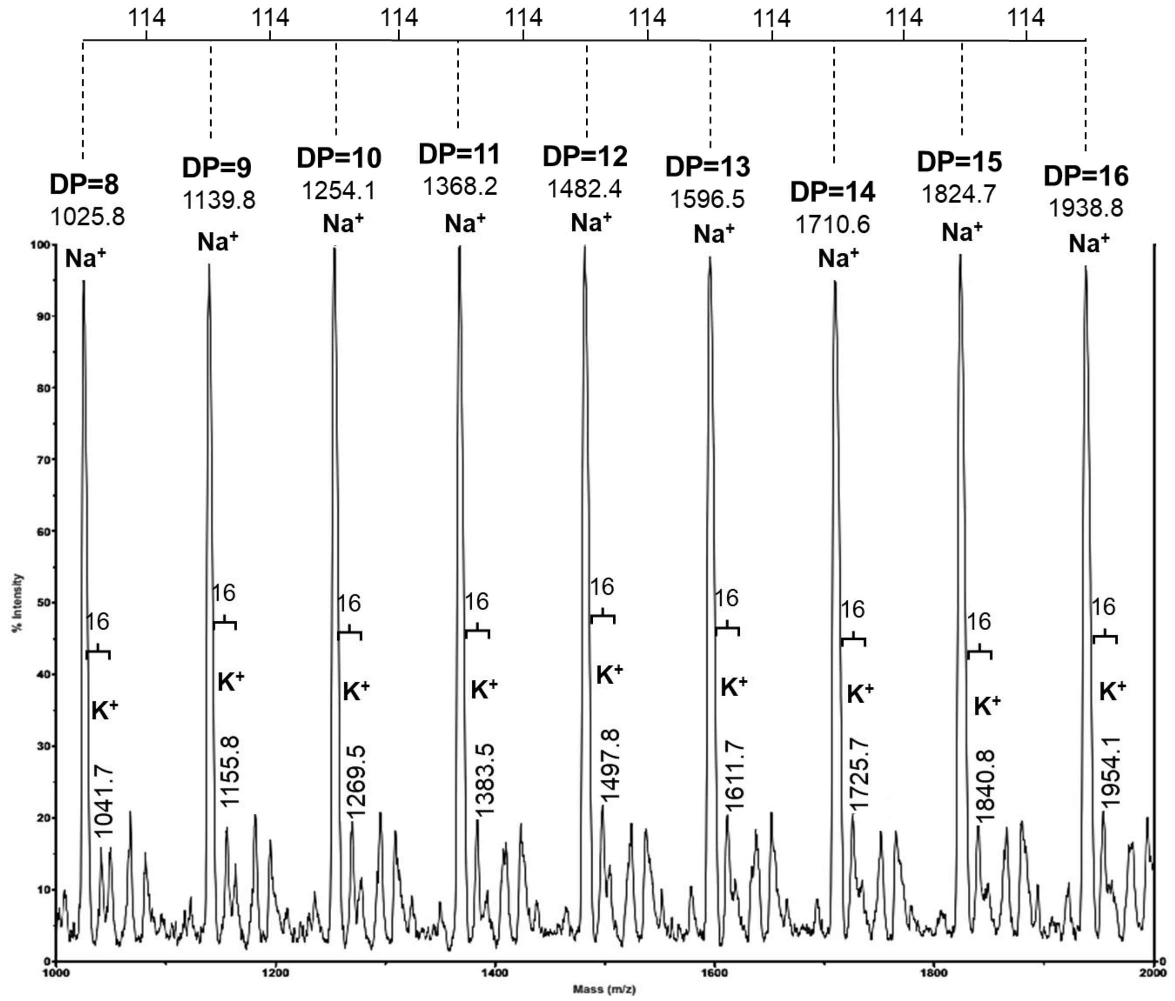

Fig. S4. MALDI-TOF spectrum (linear mode) expanded view for the 1000−2000 m/z fragments of $HOPCL_{4a10}OH$ (note: 114 and 16 are the values of the molecular weight of CL and the difference between Na^+ and K^+ (doping the same species of polymer), respectively).

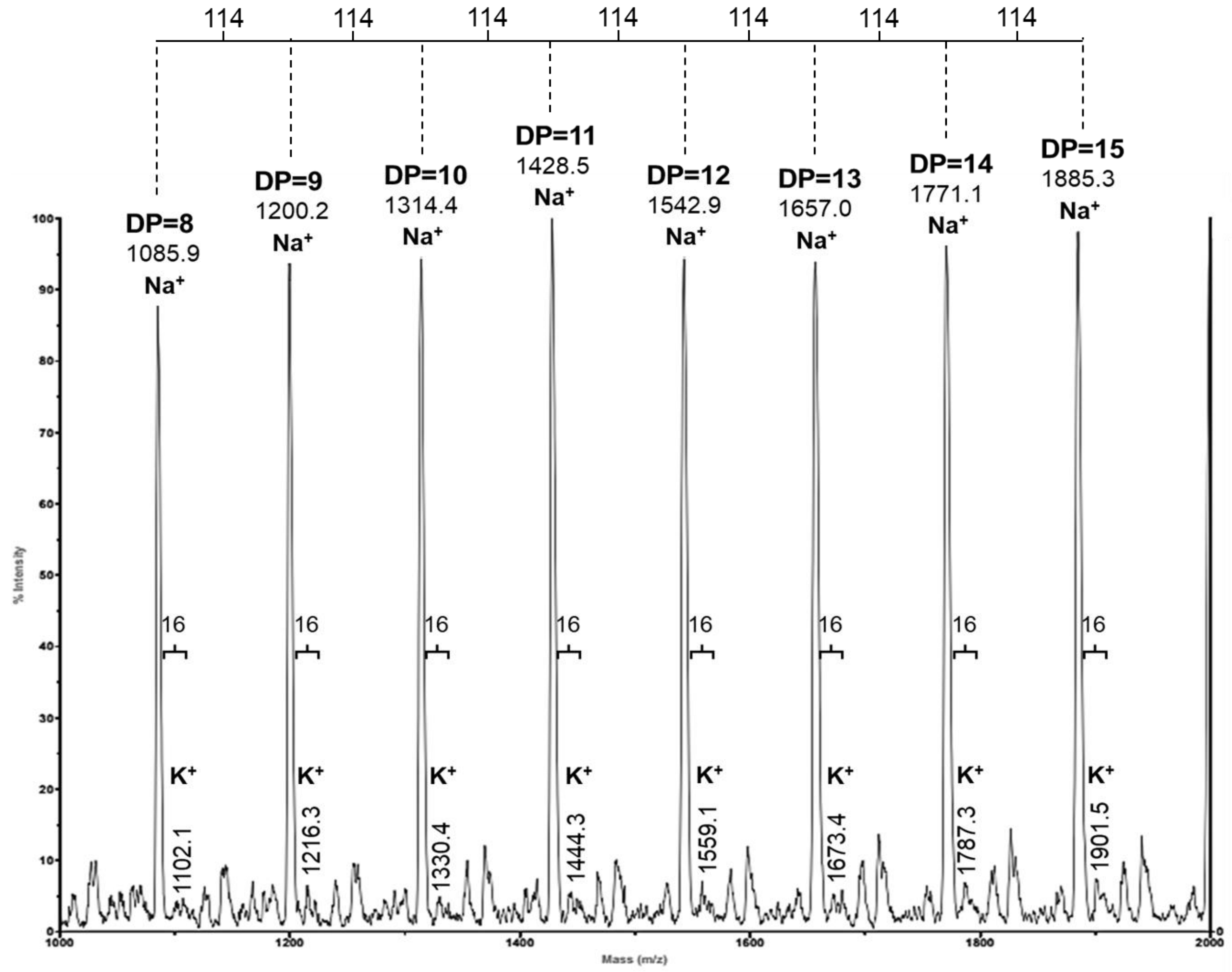

Fig. S5. MALDI-TOF spectrum (linear mode) expanded view for the 1000−2000 m/z fragments of $HOPCL_{8a10}OH$ (note: 114 and 16 are the values of the molecular weight of CL and the difference between Na^+ and K^+ (doping the same species of polymer), respectively).

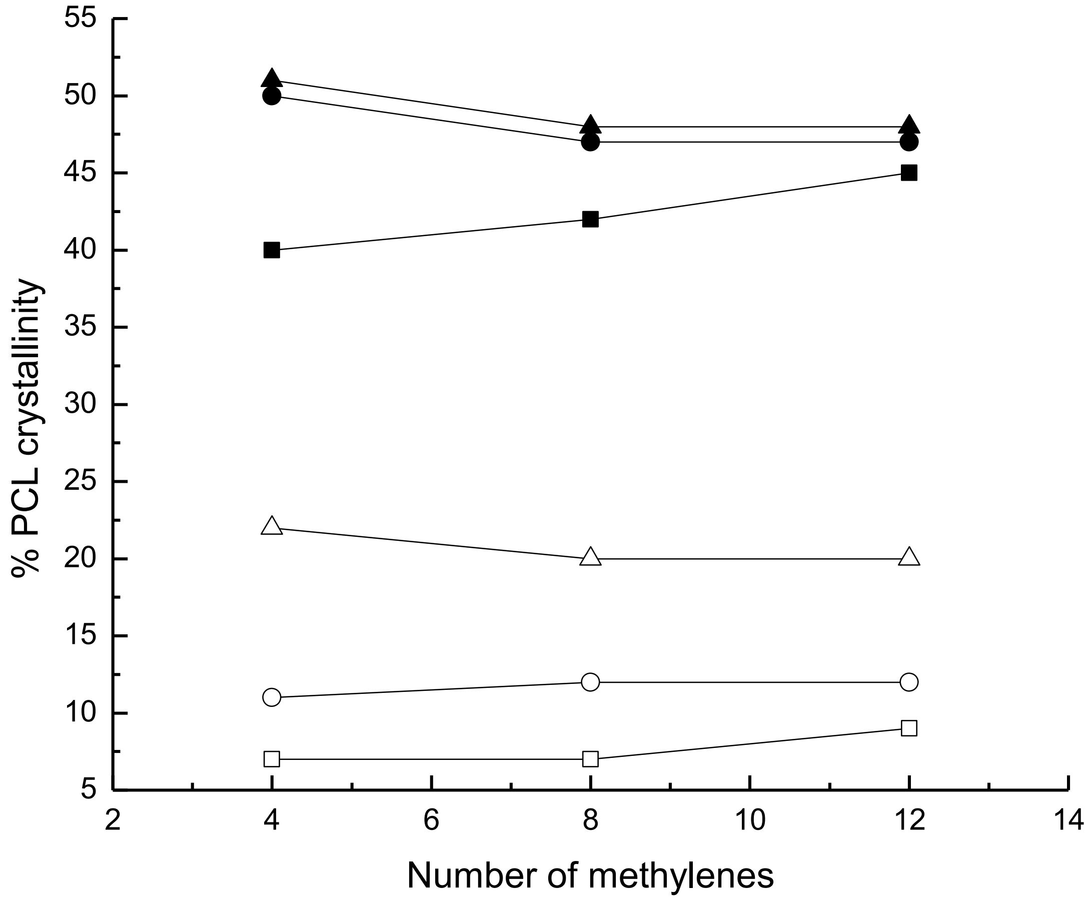

Fig. S6. Effect of number of methylenes on the crystallinity PCL for poly(ester-urethanes) (PEUs) derived from poly(ε-caprolactone) diols (HOPCLOH) and 1,6-hexamethylene diisocyanate (HDI). The PEUs derived from the macrodiols with DP=5 as initiator are indicated with open squares □, and macrodiols are indicated with filled square ■ ; PEUs derived from macrodiols with DP=10 as initiator are indicated with open circles ○, and macrodiols are indicated with filled circles ●, and PEUs derived from macrodiols with DP=15 as initiator are indicated with open triangles △, and macrodiols are indicated with filled triangle▲.

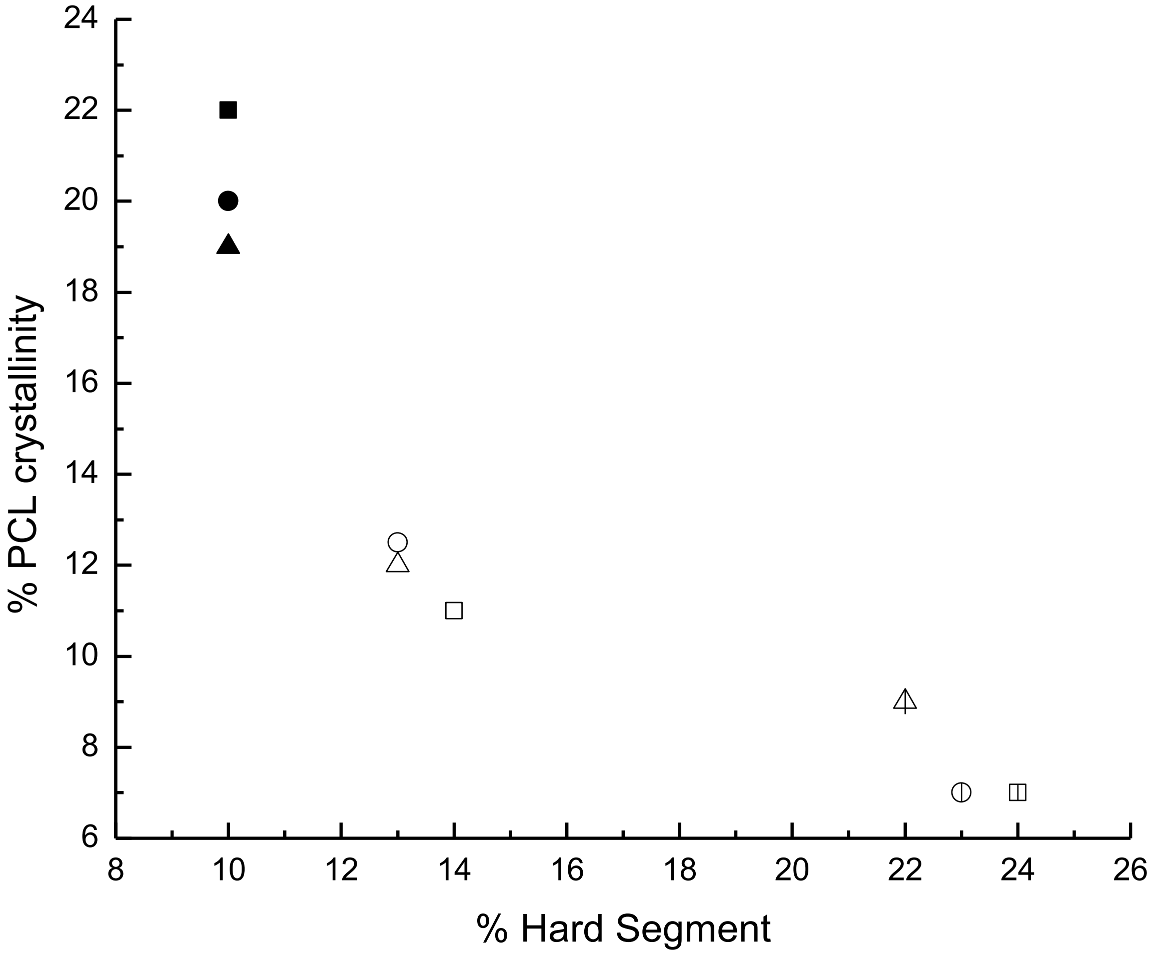

Fig. S7. Effect of Hard Segment (HS) content on the crystallinity PCL for poly(ester-urethanes) (PEUs) derived from poly(ε-caprolactone) diols (HOPCLOH) and 1,6-hexamethylene diisocyanate (HDI). The PEUs derived from the macrodiols with $HO(CH_2)_4OH$ as initiator are indicated with squares (◫ DP=5; □ DP=10, and ■ DP=15); PEUs derived from macrodiols with $HO(CH_2)_8OH$ as initiator are indicated with circles (⦶ DP=5, ○ DP=10, and ●DP=15) and PEUs derived from macrodiols with $HO(CH_2)_{12}OH$ as initiator are indicated with triangles (◬ DP=5, △ DP=10, and ▲ DP=15).

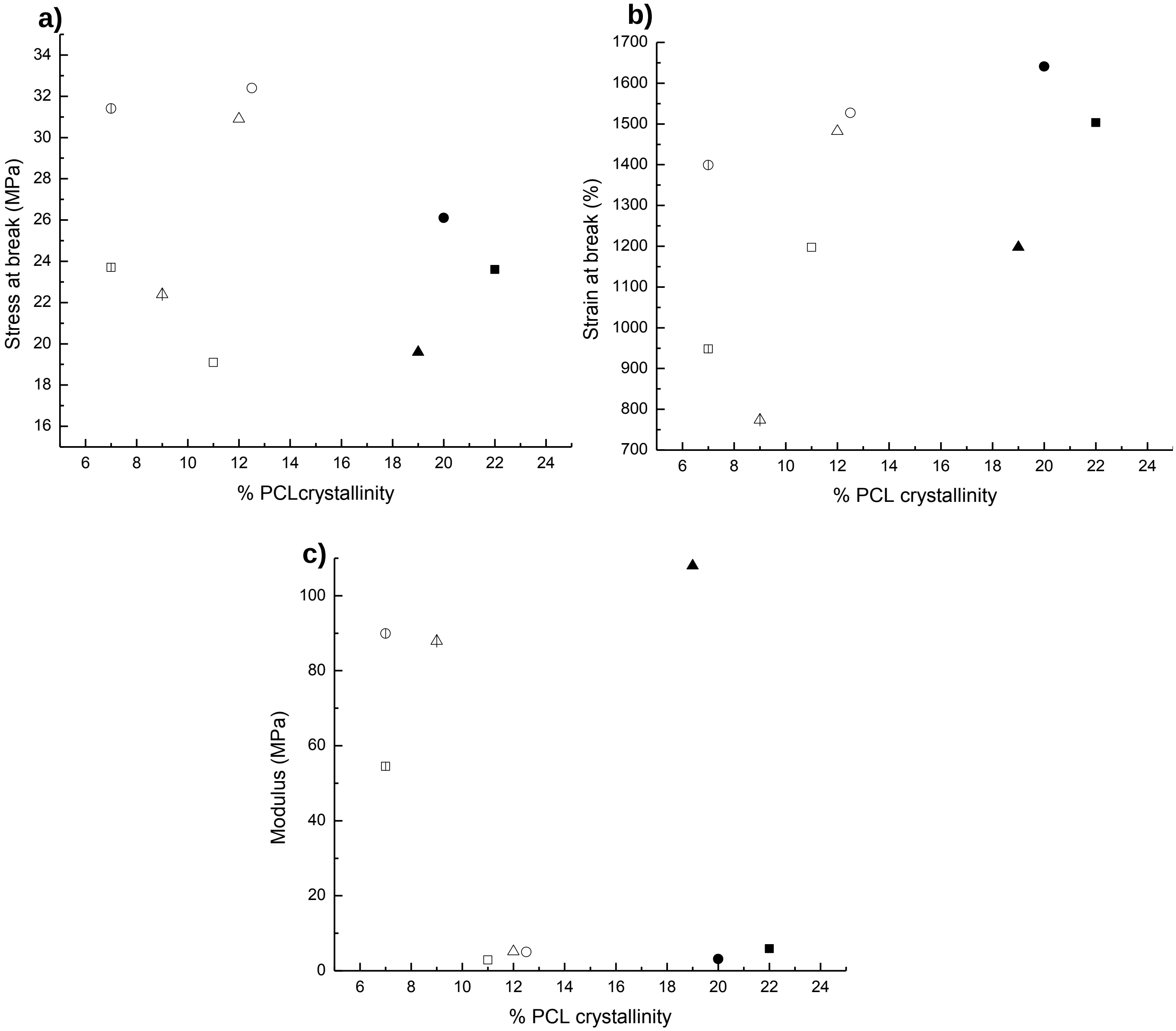

Fig. S8. Mechanical properties of the synthesized PEUs with HDI: a) stress at break, b) strain at break, and c) modulus. The PEUs derived from the macrodiols with $HO(CH_2)_4OH$ as initiator are indicated with squares (⊞ DP=5; □ DP=10, and ■ DP=15); PEUs derived from macrodiols with $HO(CH_2)_8OH$ used as initiator are indicated with circles (⦶ DP=5, ○ DP=10, and ● DP=15) and PEUs derived from macrodiols with $HO(CH_2)_{12}OH$ as initiator are indicated with triangles (⚠ DP=5, △ DP=10, and ▲ DP=15).
